# Depressive symptoms and socioeconomic status among the labor force: Evidence from China’s representative sample

**DOI:** 10.1371/journal.pone.0272199

**Published:** 2022-08-22

**Authors:** Guoying Zhang, Chang Cai, Wenxin Zou, Lu Jing, Shaolong Wu

**Affiliations:** 1 Department of Public Administration, School of Politics and Public Administration, South China Normal University, Guangzhou, China; 2 Department of Health Policy and Management, School of Public Health, Sun Yat-sen University, Guangzhou, China; 3 Sun Yat-sen Global Health Institute, Institute of State Governance, Sun Yat-sen University, Guangzhou, China; University of Southern Queensland, AUSTRALIA

## Abstract

**Objectives:**

The purpose of this paper is to describe the prevalence of depressive symptoms in the Chinese labor force; to explore the relationship between depressive symptoms and socioeconomic status among the Chinese labor force, including both the structural determinants and the intermediary determinants of health inequities; and to identify vulnerable populations who would benefit from intervention measures.

**Methods:**

Data were from the China Labor-Force Dynamics Survey (CLDS) 2016. The Center for Epidemiologic Studies Depression Scale (CES-D) was used to assess depressive symptoms. The World Health Organization’s theoretical framework of the social determinants of health was adopted to analyze the relationship between social determinants and depressive symptoms.

**Results:**

Of the participants in the research from the Chinese labor force, 17.34% were identified as having depressive symptoms. Depression was significantly related to socioeconomic factors such as hukou status (p < 0.05 in the age < 45 model), education (p < 0.01 in all five models), employment (p < 0.05 in the male model), income (p < 0.05 in all five models), and self-assessed social class position (p < 0.01 in all five models). Intermediary factors were also related to depressive symptoms, such as gender (p < 0.001 in the overall model), age (p < 0.05 in the overall model), marriage (p < 0.05 in the female model), occupational exposure (p < 0.01 in the overall model), exercise (p < 0.05 in all five models), and health insurance (p < 0.05 in the overall model). The results showed that low socioeconomic status was associated with an increased risk of depression and there were some gradient changes in the distribution of depressive symptoms in socioeconomic status.

**Conclusions:**

The findings showed that depression symptoms are significantly related to structural determinants and intermediary determinants in China’s labor force. There are some gradient changes in the distribution of depressive symptoms among people of different socioeconomic status. Low socioeconomic status is associated with increased risk of depression. Women, older people, and single and divorced people are the relative vulnerable groups in China’s labor force.

## Introduction

The World Health Organization (WHO) defines health as a state of complete physical, mental, and social well-being, and not merely the absence of disease or infirmity. Mental health is considered a very important issue in many countries [1], and depression is given the most attention among mental health problems. The major manifestations of depression include low spirits, decreased interest, slow thinking, lack of initiative, pessimism, self-blame, poor sleep, worry about illness, or experiencing multiple discomforts. Depression may also cause varying degrees of physical dysfunction, a decreased ability to live well, and cognitive decline. According to the WHO Assembly, depression is also a major contributor to global disability and should be a cause for concern. About one-fifth of the world’s population suffer from mental or behavioral disorders at some point in their lives [2, 3].

The Global Health Statistics study identified depression as the fourth biggest cause of disease burden in 1990, and that 3.7% of the world’s disability-adjusted life years (DALYs) were caused by depression [4]. In 2000, depression remained the fourth leading cause of disease burden, accounting for 4.4% of global DALYs and 12% of the world’s total years lived with disability [5]. The Global Burden of Disease Study 2010 identified mental and substance use disorders as the leading cause of YLDs worldwide, with depressive disorders accounting for 40.5% of DALYs caused by mental and substance use disorders [6].

Surveys conducted in China have estimated that 30% of men and 43% of women aged 45 years and older had depression-related symptoms in 2011 and 2012 [7], and that 23.7% of migrant workers aged 16 years and older had depressive symptoms in 2011 [8]. A survey conducted in Sichuan province reported that 12.4% of women in rural areas had depressive symptoms in 2016 [9]. A study in 2016 using data from the China Health and Retirement Longitudinal Study (CHARLS) found that more than 32.5% of the population aged 60 and above had depressive symptoms, with a higher prevalence among females than males [10].

Functional impairment, reduced quality of life, and increased mortality caused by depression place heavy burdens on both individuals and health systems [11]. Globally, the burden of disease associated with depression is similar to that of ischemic heart disease, and depression is expected to be the second most common risk factor for disability by 2020 [12, 13]. Depressed patients are more likely to have poorer physical health, worse social and role functions, worse perceptions of health conditions, and greater physical pain than patients with other chronic diseases such as hypertension, diabetes, and arthritis [14]. Depression poses a growing challenge to health systems in both developed and developing countries. A clear way to improve the mental health of the population is to develop and promote targeted preventive and therapeutic measures [15].

Current research on depression tends to focus on middle-aged and elderly people. The labor force, generally a healthier group, has attracted more attention in terms of physical health problems, including infectious diseases, occupational diseases, and injuries. Their mental health problems, on the other hand, although as serious as physical disease, have been largely ignored or forgotten, and are particularly invisible in studies on depression in China. In this study, a representative Chinese sample of the labor force aged between 15 and 64 was collected to analyze the prevalence of depressive symptoms and their influencing factors, and to discuss the underlying mechanisms and available interventive measurements.

## Theoretical framework

According to the Health Field Concept framework, direct factors influencing health can be categorized into four types—biology, environment, lifestyle, and health care organization [16]. The Health Field Concept framework has facilitated the understanding of the causes of disorders, diseases, and death. Furthermore, the distribution of these four types of factors in society is determined by socioeconomic status, the inequality of which indirectly affects the health of the population [17]. In 2010, the Commission on Social Determinants of Health (CSDH), established by the World Health Organization, developed a conceptual framework of the social determinants of health, systematically summarizing the various social factors and mechanisms that influence health inequality [18]. Our study used the WHO’s theory of health inequalities to explore the distribution of mental health problems and the social influencing factors in the Chinese labor force.

In the study of the social determinants of health, the CSDH recognized individual health to be influenced by many factors, which can be divided into two basic categories: structural determinants and intermediary determinants. Structural determinants include both socioeconomic and political context and socioeconomic position. Governance, macroeconomics, social policies, culture, and societal values are the essential elements of context. Socioeconomic position, also known as socioeconomic status, is composed of, and can be measured by, social class, gender, education, occupation, and income. Intermediary determinants are also direct factors affecting heath, indicating that material circumstances, behavior and biological factors, psychological factors, and the health system directly influence health inequality.

Intermediary determinants are influenced by socioeconomic position via the mechanism of social cohesion and social capital, determining the level and distribution of some direct health-influencing factors in the health causal chain. This marks the point at which socioeconomic status factors—such as education, occupation, and income level—shape people’s lives, influencing their exposure to risk factors that directly affect their health. However, socioeconomic position is not static, as it can be altered by governmental and public policies. Within the socioeconomic and political context, the government can formulate and implement macroeconomic and social policies and develop governance to improve social inequality.

**Fig 1:**
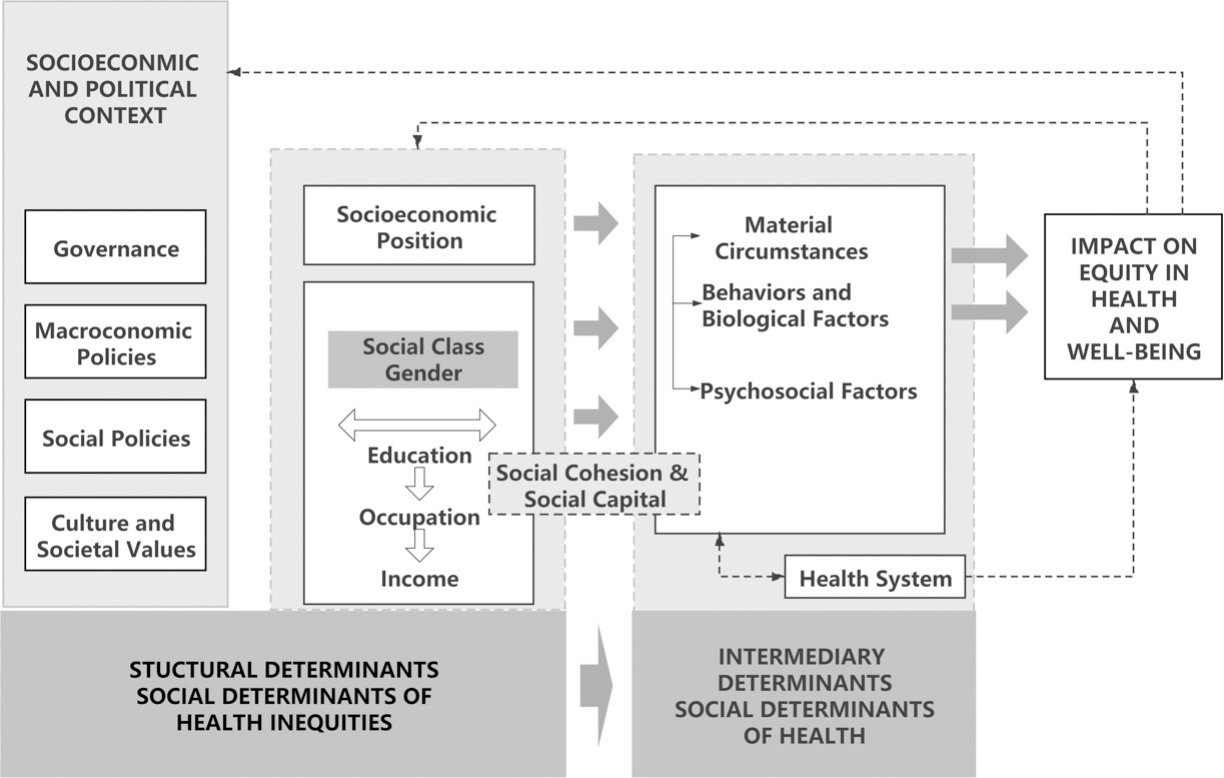
The conceptual framework of social determinants of health (SDH). Source: World Health Organization, 2010. *A conceptual framework for action on the social determinants of health*. Geneva. p. 6.

The WHO framework also shows a pathway from social determinants to health outcomes. Structural determinants such as education impact people’s health and well-being via their employment and income, as well as the associated intermediary factors. Examples of socioeconomic status, along with a range of intermediary factors, are listed in Fig 1 below. Well-educated individuals are more likely to get good jobs, which usually come with high salaries and low workplace hazards. In addition, high-income people are more likely to have high-quality housing, as well as better lifestyles and health care.

In summary, individuals with high socioeconomic status and low health risk factors tend to achieve better health outcomes. The converse is also true. In this study, the theoretical hypothesis is tested using the scale of mental health in China’s labor force.

## Methods

### Data source

The data in this research originated from the 2016 China Labor-Force Dynamic Survey (CLDS). It is publicly available, and applications can be made to download it on the website http://css.sysu.edu.cn/Data. The CLDS is a nationally representative survey designed and implemented by the Center for Social Survey, Sun Yat-Sen University. The survey is longitudinal and conducted every two years. The baseline survey was completed in 2012, and the 2016 data was the third wave. Interviews were conducted with each person from the workforce (family members aged 15–64) in the sample households. Based on the investigation of families and individuals in China’s urban and rural communities, the survey aims to monitor changes in social structure and socioeconomic status, as well as in health-related conditions.

### Sampling and sample size

The CLDS adopted multi-stage, multi-stratified Probability Proportionate to Size Sampling (PPS). It covered 29 provinces, autonomous regions, and municipalities (excluding Hong Kong, Macao, Taiwan, Tibet, and Hainan). Data collection was carried out at three levels. The first level was the community where the labor force was located, the second was families with members of the labor force aged between 15 and 64, and the third level was the individual targeted labor force. In 2016, the CLDS collected 401 community questionnaires, 14,226 family questionnaires, and 21,086 individual questionnaires. All data and samples released were fully anonymized before researchers accessed them. A total of 19,680 individuals were finally included in the analysis after excluding cases with missing values.

### Dependent variables

The Center for Epidemiologic Studies Depression Scale (CES-D) was used to measure depression in the labor force. The CES-D was prepared by the National Institute of Mental Health in 1977 and has been widely used in epidemiological investigations [19], proving its good reliability and validity [20, 21]. The scale contains 20 symptom items from four aspects of depression, namely: depressed affect, positive affect, somatic symptoms and retarded activity, and interpersonal relations [19]. Participants were required to give the frequency of the occurrence of symptoms in the previous week. Each item was measured by four scale choices (less than 1 day, 1–2 days, 3–4 days, 5–7 days), recoded respectively as 0 to 3 for the negative questions and 3 to 0 for the positive questions. The total score of this scale was between 0–60 points. The higher the total score of the scale, the more severe the depressive symptoms. Scores ≤15 were categorized as no clinical depression, 16–19 as possible depression, and ≥20 as definite depression [22, 23]. In this study, depression was divided into two categories: depression (≥16 score) and no depression (<16 score).

### Independent variables

According to the WHO’s conceptual framework of the social determinants of health, depressive symptoms as mental health outcomes are influenced by structural determinants and intermediary determinants. Following previous studies on factors affecting depressive symptoms, this study included the following variables for analysis. The focused variables for structural determinants were hukou (Chinese household registration system associated with social welfare), education, occupation, work status, income, and self-assessed social class position. The control variables for the intermediary determinants were biogenetic factors (age, gender), environment (marital status, exposure to occupational hazards), health-related behavior (smoking, drinking, exercise), and health care (health insurance).

The variables were coded as follows: Hukou was categorized into agricultural and non-agricultural. Education was categorized into four groups: primary school and below, middle school, high school, and college and above. Occupation included six subgroups: head of a public organization, professional and technical personnel, service industry personnel, manufacturing industry personnel, agriculture personnel, and other occupations. Work status was coded as five conditions: employee, employer, self-employed, no work experience, and unemployed for the past year. The annual household disposable income per capita (RMB) of respondents was broken into three groups (<10,000, 10,000–30,000, and >30,000). The self-assessed social class position was given the levels 1–10 in the questionnaire and the answers were divided into three groups: low (levels 1–3), middle (levels 4–7), and high (levels 8–10).

The respondents were divided into two age groups (<45 and ≥45 years old) and two marital statuses (married or single). We simply dichotomized exposure to occupational hazards such as smoking, drinking, exercise, and having health insurance into two groups: yes and no.

### Data analysis

The data analysis was conducted using Stata 13.0 software. First, descriptive analysis was carried out. The distributions and statistical significance of depressive symptoms among the variables were measured by a Chi-square test. Multivariable logistic regression was used to identify the factors significantly associated with depression. A total of five regression models were used to explore the relationship between depression and socioeconomic status by classifying age and gender. The subgroup analysis provided more information on the influencing factors. The level of statistical significance was established at P < 0.05 in all models.

## Results

### Characteristics of respondents

The descriptive results are shown in Table 1. Among 19,680 subjects, 47.4% were male, 56.6% were aged ≥45 years, 82.47% were married, and 78.40% had agricultural hukou. The proportion of employees, employers, self-employed people, those with no work experience, and those unemployed in the past year accounted for 28.2%, 1.37%, 39.25%, 15.13%, and 16.05%, respectively. The proportion of heads of public organizations, professional and technical personnel, service industry personnel, manufacturing industry personnel, agriculture personnel, and other occupations accounted for 1.8%, 0.42%, 19.07%, 12.14%, 32.2%, and 34.38%, respectively. The proportion of respondents with education equal to or lower than primary school was 36.03%, those with junior high school education were 34.16% of the total, those with high school education made up 17.85%, and 11.97% had college education or above. Those with an annual household disposable income per capita less than 10,000 RMB, those with between 10,000 and 30,000 RMB, and those with over 30,000 RMB accounted for 51.34%, 27.71%, and 20.96%, respectively. The majority (63.05%) of the sample considered themselves as middle class, 29.12% as low class, and 7.83% as high class. Medical insurance was held by 90.82%, respondents who smoked and drank alcohol made up 27.04% and 19.54% of the total, respectively, and 31.45% reported regularly exercising in the past month. In terms of exposure to occupational hazards, 23.14% of 17,016 respondents reported this.

**Table 1:** Descriptive statistics of the 2016 CLDS sample (N = 19680).

Variables	N	%
Gender		
Male	9,329	47.40
Female	10,351	52.60
Age		
<45 years	8,530	43.34
≥45 years	11,150	56.66
Marital status		
Married	16,231	82.47
Single	3,449	17.53
Hukou		
Agricultural	15,429	78.40
Non-agricultural	4,251	21.60
Work status		
Employee	5,550	28.20
Employer	270	1.37
Self-employed	7,724	39.25
No work experience	2,977	15.13
Unemployed for the past year	3,159	16.05
Occupation		
Head of public organization	355	1.80
Professional and technical personnel	82	0.42
Service industry personnel	3,753	19.07
Manufacturing industry personnel	2,389	12.14
Agriculture personnel	6,336	32.20
Other personnel	6,765	34.38
Education level		
Primary school and below	7,090	36.03
Middle school	6,723	34.16
High school	3,512	17.85
College and above	2,355	11.97
Income per capita (RMB)		
Less than 10,000	10,103	51.34
10,000 to 30,000	5,453	27.71
More than 30,000	4,124	20.96
Self-assessed social class position		
Low	5,731	29.12
Middle	12,409	63.05
High	1,540	7.83
Health insurance		
Yes	17,873	90.82
No	1,807	9.18
Smoke		
Yes	5,322	27.04
No	14,358	72.96
Drink		
Yes	3,845	19.54
No	15,835	80.46
Exercise		
Yes	6,189	31.45
No	13,491	68.55
Exposure (n = 17016)		
Yes	3,938	23.14
No	13,078	76.86

### Prevalence of depressive symptoms and scores of the scale

Descriptive statistics for CES-D scores of totals, dimensions, and items were reported Table 2. The mean score of the total scale was 7.345, and 17.34% of all participants reported depressive symptoms. According to the further detailed results, the scores of items on the dimension of somatic symptoms and retarded activity were the maximum values of mean and standard deviations, indicating there were more serious problems concerning body sense than affect and interpersonal relationships. The scores of mean and standard deviations on the dimension of interpersonal relations were the minimum values, indicating good interpersonal relationships and social support in China. In general, the scores of items on the dimension of positive affect were higher than that of depressed affect, indicating that the respondents in China may have a worse experience with positive affect.

**Table 2:** Descriptive statistics for each item and dimension and total score in the scale.

Item and dimension	N/Mean	%/SD	95% CI	
Total score of the total scale (0–60)	7.345	9.166	7.217	7.473
<16	16,266	82.66	82.13	83.18
≥16	3,414	17.34	16.81	17.87
Depressed affect (0–21)	2.272	3.306	2.225	2.318
I felt like I could not shake off the blues even with help from my family or friends	1.339	0.615	1.330	1.347
I felt depressed	1.468	0.679	1.458	1.477
I thought my life had been a failure	1.32	0.645	1.310	1.328
I felt fearful	1.248	0.553	1.239	1.255
I felt lonely	1.309	0.625	1.299	1.317
I had crying spells	1.208	0.497	1.201	1.215
I felt sad	1.377	0.656	1.367	1.386
Positive affect (0–12)	1.512	2.144	1.482	1.542
I felt that I was just as good as other people	1.442	0.721	1.432	1.452
I felt hopeful about the future	1.349	0.679	1.339	1.358
I was happy	1.462	0.678	1.452	1.471
I enjoyed life	1.257	0.578	1.248	1.264
Somatic symptoms and retarded activity (0–21)	3.088	3.436	3.039	3.135
I was bothered by things that usually don’t bother me	1.534	0.726	1.523	1.544
I did not feel like eating; my appetite was poor	1.504	0.729	1.493	1.514
I had trouble keeping my mind on what I was doing	1.401	0.652	1.392	1.410
I felt that everything I did was an effort	1.48	0.746	1.469	1.490
My sleep was restless	1.62	0.859	1.608	1.632
I talked less than usual	1.365	0.635	1.355	1.373
I could not get “going”	1.18	0.488	1.173	1.186
Interpersonal relations (0–6)	0.475	0.963	0.462	0.489
People were unfriendly	1.253	0.541	1.245	1.260
I felt that people disliked me	1.221	0.508	1.213	1.228

#### Socioeconomic factors affected depressive symptoms

The results of the univariate analysis are presented in Table 3. Chi-square analysis showed that the distribution differences of depressive symptoms were statistically significant among all variables except in those of marital status and occupational exposure. The prevalence of depressive symptoms was higher among women (19.57%), the high age group (19.06%), the agricultural hukou group (18.08%), the uninsured group (20.53%), non-smokers (18.01%), non-drinkers (17.95%), and non-exercisers (18.71%) than that among men (14.88%), the low age group (18.71%), the non-agricultural hukou group (14.68%), the insured group (17.03%), smokers (15.56%), drinkers (14.88%), and exercisers (14.38%), respectively. In terms of work status, those without work experience and those unemployed for the past year had the highest depression rates (19.79% and 19.47%, respectively), followed by self-employed people (18.8%), and the employee population (13.12%). Employers had the lowest depression rate (11.11%). Within occupations, respondents working in agricultural sections reported the highest depression rate (20.25%) and the professional and technical personnel had the lowest depression rate (10.98%). Unsurprisingly, the levels of depression gradually decreased the variables of education, income, and self-assessed social class position increased.

**Table 3:** Chi-square analysis of the relationship of factors with depressive symptoms.

Variables	No depression	Depression	Chi-square value	P value
Gender				
Male	7,941 (85.12)	1,388 (14.88)	75.4232	0.000
Female	8,325 (80.43)	2,026 (19.57)		
Age				
<45 years	7,241 (84.89)	1,289 (15.11)	52.5076	0.000
≥45 years	9,025 (80.94)	2,125 (19.06)		
Marital status				
Married	13,430 (82.74)	2,801 (17.26)	0.5286	0.467
Single	2,836 (82.23)	613 (17.77)		
Hukou				
Agricultural	12,639 (81.92)	2,790 (18.08)	26.9322	0.000
Non-agricultural	3,627 (85.32)	624 (14.68)		
Work status				
Employee	4,822 (86.88)	728 (13.12)	110.1832	0.000
Employer	240 (88.89)	30 (11.11)		
Self-employed	6,272 (81.20)	1,452 (18.80)		
No work experience	2,388 (80.21)	589 (19.79)		
Unemployed for the past year	2,544 (80.53)	615 (19.47)		
Occupation				
Head of a public organization	314 (88.45)	41 (11.55)	152.2058	0.000
Professional and technical personnel	73 (89.02)	9 (10.98)		
Service industry personnel	3,250 (86.60)	503 (13.40)		
Manufacturing industry personnel	2,104 (88.07)	285 (11.93)		
Agriculture personnel	5,053 (79.75)	1,283 (20.25)		
Other personnel	5,472 (80.89)	1,293 (19.11)		
Education level				
Primary school and below	5,464 (77.07)	1,626 (22.93)	245.4669	0.000
Junior high school	5,727 (85.19)	996 (14.81)		
High school	3,028 (86.22)	484 (13.78)		
College and above	2,047 (86.92)	308 (13.08)		
Income per capita (RMB)				
Less than 10,000	7,995 (79.13)	2,108 (20.87)	188.2647	0.000
10,000 to 30,000	4,654 (85.35)	799 (14.65)		
More than 30,000	3,617 (87.71)	507 (12.29)		
Self-assessed social class position				
Low	4,287 (74.80)	1,444 (25.20)	347.4223	0.000
Middle	10,654 (85.86)	1,755 (14.14)		
High	1,325 (86.04)	215 (13.96)		
Health insurance				
Yes	14,830 (82.97)	3,043 (17.03)	14.0656	0.000
No	1,436 (79.47)	371 (20.53)		
Smoke				
Yes	4,494 (84.44)	828 (15.56)	16.292	0.000
No	11,772 (81.99)	2,586 (18.01)		
Drink				
Yes	3,273 (85.12)	572 (14.88)	20.3512	0.000
No	12,993 (82.05)	2,842 (17.95)		
Exercise				
Yes	5,299 (85.62)	890 (14.38)	55.4375	0.000
No	10,967 (81.29)	2,524 (18.71)		
Exposure (n = 17016)				
Yes	3,251 (82.55)	687 (17.45)	0.1654	0.684
No	10,833 (82.83)	2,245 (17.17)		

The multivariate analysis results are reported in Table 4. In terms of statistically significant results, this research indicated that almost all variables that measured socioeconomic status demonstrated an association with depressive symptoms. The risk of depression in the non-agricultural hukou group was higher than that in the agricultural hukou group (OR = 1.230, P < 0.05) in the age < 45 model. However, the opposite was true in the age ≥ 45 model. In comparison to the labor force with primary school education or below, the risk of depression among those in the higher education level was lower in all five models, indicating the importance of education. There was a significant difference in the distribution of depressive symptoms depending on work status. Male self-employed respondents had a lower risk of depression than male employees, but the female model showed the opposite result. Therefore, it seems more difficult for women to seek self-employment in China than for men. Those engaged in agriculture were more at risk of depression than were heads of a public organization (OR = 1.980, P < 0.05) in the male model. The risk of depression for manufacturing industry personnel was lower than that for heads of a public organization (OR = 0.546, P < 0.05) in the female model, indicating greater pressure for female heads of public organizations. Respondents in both the 10,000 to 30,000 and the 30,000 and above income groups were less likely to have depression than those with an income below 10,000 in all five models. It is not surprising that income level is closely associated with depressive symptoms across most modern societies. Finally, the risk of depression in participants with high subjective identification was less than in those with low subjective identification in all five models, demonstrating that subjective class assessment can also be effectively used to explore the relationship between depression and socioeconomic status.

**Table 4:** Multivariate regression analysis of the relationship between factors and depressive symptoms among the labor force.

Independent variable	Overall (n = 17016)		Male (n = 8293)		Female (n = 8723)		Age < 45 (n = 7351)		Age ≥ 45 years (n = 9665)	
	OR	95% CI	OR	95% CI	OR	95% CI	OR	95% CI	OR	95% CI
Gender: (ref = male)	1.300***	1.165 1.450	-----	----- -----	-----	----- -----	1.215*	1.031 1.431	1.342***	1.156 1.559
Age: (ref = < 45)	1.125*	1.019 1.242	0.992	0.848 1.159	1.228**	1.078 1.400	-----	----- -----	-----	----- -----
Marital status: (ref = married)	1.129	0.998 1.277	0.981	0.810 1.188	1.205*	1.020 1.424	1.148	0.965 1.367	1.260*	1.036 1.532
Hukou: (ref = agricultural)	0.997	0.878 1.132	1.096	0.909 1.323	0.906	0.762 1.078	1.230*	1.030 1.469	0.774**	0.643 0.932
Work status: (ref = employees)										
Employer	1.027	0.672 1.570	0.937	0.556 1.587	1.16	0.562 2.396	1.204	0.747 1.942	0.488	0.175 1.361
Self-employed	0.952	0.793 1.143	0.712**	0.553 0.916	1.376*	1.048 1.806	0.831	0.640 1.080	1.095	0.843 1.422
No work experience	1.164	0.866 1.565	1.119	0.735 1.703	1.37	0.886 2.118	1.07	0.700 1.636	1.408	0.916 2.162
Unemployed for the past year	1.087	0.804 1.468	1.001	0.649 1.544	1.301	0.837 2.021	0.981	0.605 1.591	1.193	0.783 1.819
Occupation: (ref = head of public organization)										
Professional and technical personnel	0.758	0.320 1.793	0.439	0.098 1.959	1.035	0.333 3.212	0.801	0.282 2.277	0.759	0.158 3.634
Service industry personnel	0.987	0.684 1.423	1.269	0.781 2.061	0.717	0.406 1.269	1.111	0.677 1.825	0.881	0.509 1.525
Manufacturing industry personnel	0.769	0.524 1.127	1.096	0.664 1.809	0.546*	0.298 0.997	0.91	0.541 1.532	0.654	0.370 1.158
Agriculture personnel	1.178	0.787 1.763	1.980*	1.161 3.377	0.608	0.325 1.137	1.457	0.827 2.569	1	0.555 1.802
Other personnel	0.976	0.631 1.510	1.514	0.852 2.690	0.584	0.296 1.152	1.082	0.598 1.958	0.917	0.480 1.753
Education level: (ref = primary school and below)										
Middle school	0.669***	0.603 0.741	0.637***	0.545 0.744	0.715***	0.622 0.823	0.574***	0.479 0.687	0.766***	0.673 0.871
High school	0.642***	0.557 0.739	0.643***	0.525 0.787	0.658***	0.538 0.806	0.577***	0.462 0.722	0.740**	0.608 0.900
College and above	0.727**	0.603 0.876	0.794	0.605 1.041	0.684**	0.526 0.891	0.585***	0.454 0.755	0.986	0.699 1.392
Income per capita: (ref = less than 10,000)										
10,000 to 30,000	0.750***	0.661 0.850	0.792*	0.657 0.954	0.719***	0.604 0.855	0.863	0.684 1.089	0.711***	0.611 0.827
More than 30,000	0.812**	0.692 0.952	0.756*	0.601 0.951	0.893	0.710 1.122	0.87	0.672 1.126	0.773*	0.624 0.959
Self-assessed social class position: (ref = low)										
Middle	0.507***	0.465 0.553	0.583***	0.511 0.666	0.458***	0.408 0.513	0.505***	0.439 0.581	0.510***	0.457 0.570
High	0.529***	0.445 0.628	0.624***	0.487 0.799	0.453***	0.356 0.576	0.682**	0.531 0.876	0.424***	0.334 0.539
Medical insurance: (ref = yes)	1.160*	1.013 1.327	1.04	0.839 1.289	1.247*	1.047 1.485	1.223*	1.007 1.485	1.11	0.919 1.340
Smoke: (ref = yes)	0.984	0.870 1.113	0.913	0.797 1.046	1.027	0.719 1.467	0.989	0.805 1.216	0.991	0.847 1.159
Drink: (ref = yes)	1.058	0.936 1.196	1.115	0.973 1.278	0.781	0.595 1.025	0.997	0.810 1.227	1.089	0.936 1.267
Exercise: (ref = yes)	1.214***	1.101 1.338	1.225**	1.054 1.423	1.229**	1.079 1.399	1.251**	1.075 1.455	1.181*	1.039 1.343
Exposure: (ref = no)	1.177**	1.063 1.304	1.006	0.868 1.167	1.376***	1.194 1.585	1.154	0.972 1.369	1.183*	1.041 1.343
Constant	0.279	0.184 0.423	0.227	0.129 0.397	0.605	0.289 1.268	0.276	0.152 0.501	0.309	0.173 0.551

In terms of biogenetic factors, women were more likely to have depressive symptoms than men in the overall model, the < 45 model, and the ≥ 45 model, and the older respondents had a higher risk of depressive symptoms than younger people in the overall model and the female model. The results are in line with the laws of mental health. Using marital status as an indicator of social environment, single or divorced respondents had a higher risk of depression than those who were married (OR = 1.205, P < 0.05) in the female model and the ≥ 45 model. In terms of workplace environment, respondents exposed to occupational hazards were more likely to get depressive symptoms than those without exposure in the overall model, the female model, and the ≥ 45 model. A good environment, both physical and social, is again shown to be good for health. The most important direct factor for health is behavior such as smoking, drinking, and exercising. Surprisingly, in all five models, the test of the relationship between depressive symptoms and smoking and drinking was not statistically significant. It is possible that drinking and smoking as interpersonal interactions in China can alleviate psychological stress. The variable of exercising significantly affected depressive symptoms in all five models, suggesting exercise as the most simple and effective intervention. Using health insurance to represent health care, uninsured respondents had a higher risk of depressive symptoms than those insured in the overall model, the female model, and the < 45 model. Health insurance is also a cost-effective variable that can be an intervention measure.

## Discussion

Depression is a significant public health issue that causes a variety of emotional and physical problems. This study used nationally representative data on the Chinese labor force to estimate the prevalence of depressive symptoms and explore their socioeconomic influencing factors. Our findings indicated that all socioeconomic factors were significantly associated with depressive symptoms. This research spotlights the importance of socioeconomic determinants of mental health and supports the hypothesis that people with high socioeconomic status tend to achieve better health outcomes.

Our study reported the prevalence of depressive symptoms in China’s labor force at 17.34% in 2016. As the working population tends to be healthier than other population groups, depressive symptoms should be relatively lower than among other groups. According to a nationwide survey in China in 2018, the prevalence of depressive symptoms among informal and formal employees were 25.5% and 19.3%, respectively [24]. The prevalence of depressive symptoms in other vulnerable populations, such as suicide attempters 60.4% [20], postpartum mothers 30% [25], female migrant workers 25.58% [26], and middle-aged and older Chinese people 18.6% [27], also supports the case for the relative health of the labor force. However, health inequality still exists in the subpopulations of China’s labor force, and is determined by socioeconomic status.

As with most populous developing countries, the urban-rural dual structure is the basis of socioeconomic status in China. In general, urban residents tend to enjoy better living conditions, healthier lifestyles, and lower health risks than rural residents. An exploration of the relationship between depression and hukou status in Chinese people aged 45 and over showed the level of depressive symptoms to be significantly higher in the population of agricultural hukou [28]. This is in accordance with our research and supports the hypothesis regarding the effects of socioeconomic status on health. However, the opposite results have also been found among those aged less than 45 years. Since this has rarely appeared in previous literature, we infer that it may be caused by fierce market competition and social stress in urban areas. The young labor force tends to face much stronger market competition in urban areas than in rural areas. After middle age, people in rural areas tend to have poorer social security than those living in urban areas.

A lower education level was significantly associated with depressive symptoms, consistent with previous research. It may be inferred that those with higher education levels have better perceptions of stress and self-regulation and formed a regulation mechanism against external pressure during their education. Furthermore, they have a better sense of pressure control, allowing them to adopt better strategies in the face of obstacles [29]. Comparably, those with low education levels may lack coping skills when facing challenges in life [30–32]. Education is a part of an individual’s resources and is more stable than other factors, such as training, that can affect occupation and income.

Using industries and employment to measure occupation, this study found some gender differences in depressive symptoms. Respondents with low-level occupations had a higher risk of depression in our research, consistent with relevant previous studies [33]. In terms of the distribution of depressive symptoms among the self-employed and employees, women and men had diametrically opposite mental health effects. The results suggest that we should pay more attention to the mental health of low-level occupation groups in China, especially self-employed women.

In the market economy, income is the most important indicator of economic status for the labor force, although some who are economically inactive are financially supported by their rich families. This paper attempted to accurately measure the relative economic position of the labor force through annual household disposable income per capita, which is similar to national disposable income per capita. Previous studies have confirmed that the risk of depression in high-income groups was lower than in low-income groups [34]. As an important economic resource for individuals, income provides risk protection and enables people to seek high-quality psychotherapy services in the event of emotional problems [35]. Although the social security system is helpful in reducing the income gap between different social classes, the cost of policy intervention can be enormous.

In terms of self-assessed social class status, the results of this study also support the hypothesis regarding the impact of socioeconomic status on health. Individuals with a higher self-assessed social class position had less risk of depressive symptoms. Previous studies have shown that individuals with higher socioeconomic status may have a lower risk of depression [36–38]. Individuals with a higher social class position have more resources, including income, education, and social support. When these individuals are faced with stressful events or low mood, they have more resources and coping styles to alleviate the degree of depression. Self-assessed social class position not only refers to the current overall self-assessment of socioeconomic status, but also reflects past living standards, including health levels. It is possible that good physical and mental health in the past affects a person’s current mental health.

This study also reported the effect of biogenetic factors. The risk of depression in older people was higher than in younger people, consistent with a study conducted in the Norwegian population. As age increases, an individual’s physical health becomes more fragile and more vulnerable to disease and damage to health, which may result in being unable to adapt to certain job requirements [39, 40]. Despite great progress in women’s rights and welfare in China, the female respondents were still more likely to suffer depressive symptoms than were the male respondents. With further social and economic development, we hope conditions could improve rapidly.

The social support provided by marriage suggests that the loss of a spouse is often associated with an increased risk of depression due to loss of financial, emotional, physical, and instrumental support. On one hand, the social relations and habit patterns of recently single or divorced people are changed, potentially making them more vulnerable to stressful events. On the other hand, social resources are more commonly available to married than to divorced people, reducing the severity of emotionally damaging events for married people and making it easier for them to recover from stress and despair [41]. Occupational hazards are a direct influencing factor, as labor force workers may experience increased psychological stress due to health damage caused by occupational exposure. Additionally, occupational hazards can cause physical damage, resulting in a decline in labor income due to the cost of treatment, and may thus generate a higher risk of depression [42, 43]. Environmental factors, although not immutable, are also very difficult to change or to intervene in over a short time.

The prevalence of depression in non-smoking and non-drinking individuals was higher than in those who smoked and drank alcohol. This may be due to the fact that smoking and drinking can have an alleviating effect on depressive symptoms [44, 45]. Whether or not respondents participated in exercise significantly affected the level of depressive symptoms. Literature has shown that exercise is an evidence-based treatment for depression and its anti-depression effect can be compared with drugs and other measures. Moreover, the treatment effect is more obvious in patients with major depression [46, 47]. Within their social circles, individuals are afraid of being discriminated against or stigmatized if they show signs of depression. Exercise is a viable treatment that can be used by most depressed patients and does not bring a negative social stigma. As an integral part of an individual’s lifestyle, exercise can alleviate stress and reduce the risk of depressive symptoms [48].

The results show that a lack of health insurance may also increase the risk of depression, suggesting that being uninsured is a risk factor for depression [49, 50]. As an important economic aspect of an individual’s medical services, health insurance determines to some extent whether one can actively seek medical services. Purchasing health insurance can increase one’s sense of security and reduce the financial and psychological stress of receiving medical treatment. Uninsured individuals who suffer health issues may be unable to afford medical expenses and therefore either reduce or not seek medical services at all, which may have adverse effects on their mental health [51]. In recent years China has almost achieved universal health insurance nationwide, but the health coverage of migrant workers still poses a challenge to be overcome.

The policy implications of this study aim to find the vulnerable groups and the intervention field of China’s labor force. Based on the multivariate results, China’s public policies should focus more on the mental health problems of women, older people, and single and divorced people in the labor force to improve health inequalities. It would also be beneficial to expand health insurance coverage, promote education popularization, and reduce occupational hazard exposure to improve the mental health of China’s labor force.

Several limitations of this study should be noted. First, the CES-D scale is a self-report inventory that uses a continuous scale to define the levels of depressive symptoms; participants may be biased in the reporting process and their judgment of depressive symptoms is not as accurate as a clinical diagnosis. However, the literature suggests that the CES-D scale is efficient and effective in assessing the symptoms of depression in the population. Second, the study was a cross-sectional survey, and we were unable to determine the causal relationship between depression and the above factors. However, due to the fact that this study was a large sample investigation, the results provide relevant suggestions for the relationship between depression and its influencing factors. In order to further explore the role of various risk factors in the development of depression, we need to conduct a longitudinal research study.

## Conclusion

This study explored the socioeconomic factors that affect the mental health status of the labor force in China. The results showed that low socioeconomic status is associated with increased risk of depression. Depression symptoms are significantly related to structural determinants, such as hukou status, education, occupation, employment, income, and self-assessed social class position. Symptoms of depression are also related to intermediary determinants, such as age, gender, marriage, health insurance, exercise, and occupational hazard exposure. Women, older people, and single and divorced people are the relative vulnerable groups in China’s labor force. Although the relationship between mental health and socioeconomic status is not necessarily a causal relationship, it shows that there are some gradient changes in the distribution of depressive symptoms among people of different socioeconomic status.

## Supporting information

S1 File CLDS-questionnaire. (DOC)

S2 File The data in this research originated from the 2016 China Labor-Force Dynamic Survey (CLDS). (ZIP)
