# Supplementary material for: Depressive symptoms and socioeconomic status among the labor force: Evidence from China’s representative sample
Source: PLoS One. 2022 Aug 22;17(8):e0272199. doi: 10.1371/journal.pone.0272199 (PMC9394851; doi:10.1371/journal.pone.0272199)
Supplement: S1 File — (DOC) [file pone.0272199.s001.doc]

**2016年中国劳动力动态调查**

**劳动力个体新增问卷**

**（20160601版本）**

1. **问卷编号:** [____|____|____|____|____|____|____|____|____|____|____|____]

**（说明：个体问卷编号为家庭问卷编号加上个体的家庭成员代码）**

**2.被访者家庭地址**

_____省（自治区、直辖市）、_____市（地区、盟、州）、_____县(区、旗、县级市)

________街/乡镇_________村/居,________门牌号

**邮政编码** ________________

**3.被访问对象姓名、联系方式：**

| 姓名 | 固定电话号码 | 手机号码 | Email |
| --- | --- | --- | --- |
|  |  |  |  |

**4.访问开始时间**：___月___日____时____分; 结束时间：____时_____分

**5.访问总长度：**___________ (分钟)

**6.访问员（签名）**___________________

访问员注意请根据以下条件对家庭成员中的劳动力个体进行甄别筛选：

1. 年龄是15-64岁的人；

② 65岁以上并且仍然在工作的人；

③ 所有问题“拒绝回答”填写99997，“不适用”填写99998，“不清楚”填写99999）

**(一)基本情况**

【CAPI】加载成员代码、姓名、性别，与101的关系、出生年月、婚姻类型变量

访问员按照此信息找到对应的被访者。

【data】生成姓名，性别，与101的关系，出生年月，婚姻状况，家庭地址数据，现在户口地数据

**I1.1** 您的出生年月是：*加载家庭数据*年*加载家庭数据*月，请问正确吗？

1.正确（跳至I1.2） 2.不正确

**I1.1.1** 您正确的出生年月应该是______年_____月

**I1.2** 您的婚姻状况是：*加载家庭数据*，请问正确吗？

1.正确（跳至I1.2.2） 2.不正确

**I1.2.1** 您正确的婚姻状况是：

1.未婚 2.初婚 3.再婚 4.离异 5.丧偶 6.同居

【data】 if I1.2==2, 更新新的婚姻状况

【CAPI】 若更新的婚姻状况=2 3 4 5,则问I1.2.2：

**I1.2.2** 您的初婚发生在哪一年（初婚指第一次婚姻）？ ______年

【CAPI】若被访者为男性,则继续询问I1.2.3和I1.2.4：

**I1.2.3** 您初婚的时候，您家总共花了多少钱？_______元（包括为结婚而建房或买房，彩礼、婚宴费等）

**I1.2.4** 在这笔花费中，多少是由您的长辈给的？_______元

**I1.3.1** 请问您正确户口所在地是在：______省（直辖市）______市______县/区

【data】 生成被访者现在的户口所在地变量（包括省市县）hkpro,hkcit,hkcon

【CAPI】 If 所选县区等于调查点县区，则询问I1.3.2，否则跳至I1.3.3

【data】 if 所选县区不等于调查点县区，自动赋值I1.3.2=4

**I1.3.2**您的户口是在：

1.本村/居委会 2 本乡镇（街道）其他村居委会

3 本县（县级市、区）其他乡镇街道 4 本县区以外

**I1.3.3**您有几位兄弟姐妹？_________位（如果为独生子女，请填0）

**I1.4** 请访问员确定，被访者父亲是否是下列哪位成员：【加载家庭成员】 2.不在家庭成员内

【CAPI】 if I1.4==2，则询问I1.4.1-I1.4.9.w16，否则跳至I1.5

**I1.4.1** 您父亲的出生年是_______年

**I1.4.2** 您父亲是否健在？ 1.是（跳至I4.4） 2.否

**I1.4.3**  您父亲是哪一年去世的？_______年

**I1.4.4**  您父亲的户口类型是？（如果父亲去世，填写去世前的户口）

1.农业户口 2.非农户口 3居民户口（之前是农业户口）

4.居民户口（之前是非农户口） 5其他（请注明）_______

**I1.4.5** 您的父亲是否是中共党员？ 1.是 2.否 99999.不清楚

**I1.4.6** 您父亲的教育程度是(见编码3)：_________

1.未上过学 2.小学/私塾 3.初中

4.普通高中 5.职业高中 6.技校

7.中专 8.大专 9.大学本科

10.硕士 11.博士 12. 其他（请注明）___

99999.不清楚

**I1.4.7** 您父亲目前的职业是什么？（如果父亲退休或已去世，填写退休前或去世前最后的职业）

_____________（请尽可能详细地描述其工作内容、岗位、工种或职责）

如：办公室文员，流水线装配工，在街头摆摊卖水果等

**I1.4.8.w16**您父亲目前工作的单位或公司属于什么行业（见编码37）？

1.农、林、牧、渔业 2.采掘业 3.制造业

4.电力、煤气及水的生产和供给业 5.建筑业 6.质勘查业、水利管理业

7. 交通运输、仓储及邮电通信业 8. 批发和零售贸易、餐饮业 9.金融保险业

10.房地产业 11.社会服务 12. 卫生、体育和社会福利业

13.教育、文化艺术和广播电影电视业 14 科学研究和综合技术服务业

15. 国家机关、党政机关和社会团体 99. 其他行业 99998.不适用 99999不清楚

**I1.4.9.w16**您父亲目前工作的单位类型是什么？

【有单位】

1.党政机关、人民团体、军队 2.国有/集体事业单位 3.国营企业

4.集体企业 5.村居委会等自治组织 6.民营、私营企业

7.外资、合资企业 8 民办非企业、社团等社会组织

9.个体工商户（包括登记过的个体工商户或未登记的各类店主）

【无单位】

10.务农：农林牧副渔业生产（如种地、养殖鸡鸭水产等）

11.自由工作者（自由职业者，零散工，摊贩，无派遣单位的保姆，自营运司机，手工工匠

**I1.5** 请访问员确定，被访者母亲是否是下列哪位成员：【加载家庭成员】 2.不在家庭成员内

【CAPI】 if I1.5==2，则询问I1.5.1-I1.5.9.w16，否则跳至I1.27 .w16

**I1.5.1** 您母亲的出生年是_______年

**I1.5.2** 您母亲是否健在？ 1.是（跳至I1.5.4 ） 2.否

**I1.5.3** 您母亲什么时候去世的？_______年

**I1.5.4** 您母亲的户口类型是？（如果母亲去世，填写去世前的户口）

1.农业户口 2.非农户口 3居民户口（之前是农业户口）

4.居民户口（之前是非农户口） 5其他（请注明）_______

**I1.5.5** 您的母亲是否是中共党员？ 1.是 2.否 99999.不清楚

**I1.5.6** 您母亲的教育程度是（见编码3）：_________

1.未上过学 2.小学/私塾 3.初中

4.普通高中 5.职业高中 6.技校

7.中专 8.大专 9.大学本科

10.硕士 11.博士 12. 其他（请注明）___

99999.不清楚

**I1.5.7** 您母亲目前的职业是什么？（如果母亲退休或已去世，填写退休前或去世前最后的职业）

_____________（请尽可能详细地描述其工作内容、岗位、工种或职责）

如：办公室文员，流水线装配工，在街头摆摊卖水果等

**I1.5.8.w16**您母亲目前工作的单位或公司属于什么行业（见编码37）？

1.农、林、牧、渔业 2.采掘业 3.制造业

4.电力、煤气及水的生产和供给业 5.建筑业 6.质勘查业、水利管理业

7. 交通运输、仓储及邮电通信业 8. 批发和零售贸易、餐饮业 9.金融保险业

10.房地产业 11.社会服务 12. 卫生、体育和社会福利业

13.教育、文化艺术和广播电影电视业 14 科学研究和综合技术服务业

15. 国家机关、党政机关和社会团体 99. 其他行业 99998.不适用 99999不清楚

**I1.5.9.w16**您母亲目前工作的单位类型是什么？

【有单位】

1.党政机关、人民团体、军队 2.国有/集体事业单位 3.国营企业

4.集体企业 5.村居委会等自治组织 6.民营、私营企业

7.外资、合资企业 8 民办非企业、社团等社会组织

9.个体工商户（包括登记过的个体工商户或未登记的各类店主）

【无单位】

10.务农：农林牧副渔业生产（如种地、养殖鸡鸭水产等）

11.自由工作者（自由职业者，零散工，摊贩，无派遣单位的保姆，自营运司机，手工工匠）

**I1.27 .w16**您14岁时，父亲的主要职业具体是？_____________（选择：职业大类）

1.国家机关、党群组织、企业、事业单位负责人

2.专业技术人员

3.办事人员和有关人员

4.商业、服务业人员

5.农、林、牧、渔、水利业生产人员

6.生产、运输设备操作人员及有关人员

7.不便分类的其他从业人员

99998.不适用

99999.不清楚

**I1.28 .w16**您14岁时，父亲工作的单位类型是什么？

【有单位】

1.党政机关、人民团体、军队 2.国有/集体事业单位 3.国营企业

4.集体企业 5.村居委会等自治组织 6.民营、私营企业

7.外资、合资企业 8 民办非企业、社团等社会组织

9.个体工商户（包括登记过的个体工商户或未登记的各类店主）

【无单位】

10.务农：农林牧副渔业生产（如种地、养殖鸡鸭水产等）

11.自由工作者（自由职业者，零散工，摊贩，无派遣单位的保姆，自营运司机，手工工匠等）

99998.不适用

99999.不清楚

**I1.29.w16**您14岁时，母亲的主要职业具体是？_____________（选择：职业大类）

1.国家机关、党群组织、企业、事业单位负责人

2.专业技术人员

3.办事人员和有关人员

4.商业、服务业人员

5.农、林、牧、渔、水利业生产人员

6.生产、运输设备操作人员及有关人员

7.不便分类的其他从业人员

99998.不适用

99999.不清楚

**I1.30.w16**您14岁时，母亲工作的单位类型是什么？

【有单位】

1.党政机关、人民团体、军队 2.国有/集体事业单位 3.国营企业

4.集体企业 5.村居委会等自治组织 6.民营、私营企业

7.外资、合资企业 8 民办非企业、社团等社会组织

9.个体工商户（包括登记过的个体工商户或未登记的各类店主）

【无单位】

10.务农：农林牧副渔业生产（如种地、养殖鸡鸭水产等）

11.自由工作者（自由职业者，零散工，摊贩，无派遣单位的保姆，自营运司机，手工工匠等）

99998.不适用

99999.不清楚

**I1.31.w16**您14岁时，父母的婚姻状况是：

1.未婚 2.初婚 3.再婚 4.离异 5.丧偶 6.同居

**I1.6** 您的政治面貌是： 1.中共党员，入党时间：________年

2.民主党派，入党时间：________年

3.群众

**I1.7** 您是否参过军？ 1.是 2.否**(跳到I1.8)**

**I1.7.1**入伍时间:__________年； 退伍时间：__________年

**I1.8** 您是否懂外语？ 1.是，包括____ ____ ____语种（如果超过三种，则选最熟练的三个）

2.否**（跳至I1.8.3）**

【CAPI】if I1.8的第二，第三不是空的则回答I1.8.1，否则跳至I1.8.2

**I1.8.1**您最熟练的外语是什么?

1. 英语 2.日语 3.俄语 4.法语 5.德语

6.西班牙语 7.韩语 8.阿拉伯语 9.其他（请注明）______

**I1.8.2** 您最熟练的外语的熟练程度是？

1.很难进行听说读写 2.勉强能听说读写

3.能比较熟练地听说读写 4.能非常熟练地听说读写

**I1.8.3** 请问您上班/上学时，**主要**使用的语言是？

“普通话”指国家颁布的现代标准汉语，如中央广播、电视新闻的语音语调。“本地方言”指调查所在地的方言，如果本地方言是普通话则优先选择普通话。“老家方言”针对流动人口而言的、指流动人口老家使用的方言。

1.普通话 2.本地方言 3.老家方言 4.其他（请注明）____

**I1.8.4** 请问您下班/放学后，**主要**使用的语言是？

“普通话”指国家颁布的现代标准汉语，如中央广播、电视新闻的语音语调。“本地方言”指调查所在地的方言，如果本地方言是普通话则优先选择普通话。“老家方言”针对流动人口而言的、指流动人口老家使用的方言。

1.普通话 2.本地方言 3.老家方言 4.其他（请注明）____

**I1.9** 您父亲出生时的户口性质是？ 1.农业户口 2.非农户口 99998.不适用

**I1.11** 您母亲出生时的户口性质是？ 1.农业户口 2.非农户口 99998.不适用

**I1.13** 您出生时的户口性质是？

1.农业户口 2.非农户口 3居民户口 4.其他（请注明）_______

**I1.14** 您目前的户口性质是？

1.农业户口 2.非农户口 3居民户口（之前是农业户口）

4.居民户口（之前是非农户口） 5其他（请注明）_______

**【CAPI】**

if I1.14==2 3 4则需要回答I1.14.1和I1.14.2，否则跳至I1.15

**I1.14.1** 您的非农或居民户口是哪一年获得的？

记录：[___|____|____|____]年 2.一出生就是

**I1.14.2** 您的非农户口是什么途径获得的（见编码4）？

1.升学 2.参军 3.招工或顶替 4.提干或转干（部队转业等）

5.婚姻 6.随父母或子女转 7.家里土地被征用

8.购买户口 9.买房入户 10.村改居 11.积分入户

12.户口改革，当地不再有农业户口 13.一出生就是 14.其他（请注明____）

【CAPI】如果I1.3.2>=3则需要询问I1.15，否则跳至I1.16

**I1.15** 您离开您的户口所在地超过半年吗？ 1.有 2.否

**I1.16**  您的户口是否迁移过？ 1是 2否**(**跳至I1.19.0**)**

“迁移”是指物理空间上的变化，即被访者的户口登记地从一个地方迁到另一个地方

**I1.16.1** 您的户口一共迁移过______次.

【CAPI】根据I1.16.1填答的次数，循环询问I1.17.1.1至 I1.17.1.2

**I1.17.1.1** 您的*第一次*户口迁移是发生在哪一年？__________年

**I1.17.1_2** 您的*第一次*户口迁移的迁移原因是什么（见编码5）？

1.务工经商 2.工作调动 3.升学/毕业

4.分配录用 5.家属随迁 6.拆迁搬家

7.婚姻迁入 8.转干 9.参军

10.支内/支边 11.上山下乡 12.其他（请注明）_______

**I1.19.0** 您有下列哪些医疗保险/保障？

**I1.19.1** 城镇职工基本医疗保险 1.是 2.否 99999.不清楚

城镇职工基本医疗保险通过**用人单位**和**个人**缴费，建立医疗保险基金，参保人员患病就诊发生医疗费用后，与医疗保险经办机构给与一定的经济补偿，以避免或减轻劳动者因患病、治疗等所承受的经济风险。

**I1.19.2** 城镇居民基本医疗保险 1.是 2.否 99999.不清楚

城镇居民基本医疗保险是社会医疗保险的组成部分，采取以政府为主导，以居民个人（家庭）缴费为主，政府适度补助为辅的筹资方式，按照缴费标准和待遇水平相一致的原则，为城镇居民提供医疗需求的医疗保险制度。

**I1.19.3** 新型农村合作医疗 1.是 2.否 99999.不清楚

新型农村合作医疗，简称“新农合”，是指由政府组织、引导、支持，农民自愿参加，个人、集体和政府多方筹资，以大病统筹为主的农民医疗互助共济制度。

**I1.19.40** 城乡居民医疗保险 1.是 2.否 99999.不清楚

城乡居民医疗保险是合并城镇居民基本医疗保险和新型农村合作医疗而建立起来的城乡一体的医疗保险制度。参保对象一般包括包括农村居民、城镇非从业居民、国家和我省规定的其他人员(不含灵活就业人员)

**I1.19.4** 公费医疗或劳保医疗 1.是 2.否 99999.不清楚

公费医疗指国家为保障**国家工作人员**而实行的、通过医疗卫生部门按规定向享受人员提供免费医疗及预防服务的一项社保制度。[劳保](http://baike.baidu.com/view/53679.htm)医疗即企业单位的医疗待遇办法，按公费医疗和劳保医疗规定，职工就医时除交挂号费外，其他医疗费用全部由[企业负担](http://baike.baidu.com/view/4502435.htm)。

**I1.19.5** 单位补充医疗保险 1.是 2.否 99999.不清楚

单位补充医疗保险是基本医疗保险的有力补充，是在单位和职工参加统一的基本医疗保险后，由单位或个人根据需求和可能原则，增加医疗保险项目，来提高保险保障水平的一种补充性保险。

**I1.19.6** 公务员医疗补助 1.是 2.否 99999.不清楚

公务员医疗补助是**在城镇职工基本医疗保险制度的基础上**，对公务员实施的**补充**医疗保障，主要用于补助公务员在基本医疗保险用药目录、诊疗项目和医疗服务设施标准范围内的住院医疗费用中个人自付超过一定数额的部分。

**I1.19.7** 商业医疗保险 1.是 2.否 99999.不清楚

商业医疗保险是医疗保障体系的组成部分，单位和个人自愿参加。指**由保险公司经营**的，赢利性的医疗保障。消费者依一定数额交纳保险金，遇到重大疾病时，可以从保险公司获得一定数额的医疗费用。

**I1.19.8** 其他医疗保险/保障，请注明_____________

**I1.20** 目前，您有没有参加下列养老保险（包括单位退休制度）？

**I1.20.1** 单位退休金/机关事业单位养老保险 1.是 2.否 99999.不清楚

**I1.20.2** 城镇职工基本养老保险 1.是 2.否 99999.不清楚

企业职工基本养老保险是国家根据法律、法规强制建立和实施的一种社会保险制度。在这一制度下，用人单位和劳动者必须依法缴纳养老保险费，在劳动者达到国家规定的退休年龄或因其他原因而退出劳动岗位后，社会保险经办机构依法向其支付养老金等待遇，从而保障其基本生活。

**I1.20.3** 城镇居民社会养老保险 1.是 2.否 99999.不清楚

城镇居民社会养老保险：年满16周岁（不含在校学生）、**不符合职工基本养老保险参保条件**的城镇非从业居民，可以在户籍地自愿参加城镇居民养老保险。

**I1.20.4** 新型农村社会养老保险 1.是 2.否 99999.不清楚

新型农村社会养老保险，称为“新农保”，是继取消农业税、农业直补、新型农村合作医疗等政策之后的又一项重大惠农政策。采取个人缴费、集体补助和政府补贴相结合，其中中央财政将对地方进行补助，并且会直接补贴到农民头上。

**I1.20.50** 城乡居民养老保险 1.是 2.否 99999.不清楚

城乡居民养老保险是合并新型农村社会养老保险和城镇居民社会养老保险而建立起来的养老保险制度。年满16周岁（不含在校学生），非国家机关和事业单位工作人员及不属于职工基本养老保险制度覆盖范围的城乡居民，可以在户籍地参加城乡居民养老保险。

**I1.20.5** 企业年金（企业补充养老保险） 1.是 2.否 99999.不清楚

企业年金（企业补充养老保险）是指企业及其职工在**依法参加基本养老保险的基础上，自愿建立的补充养老保险制度。**

**I1.20.6** 商业性养老保险 1.是 2.否 99999.不清楚

商业养老保险是以获得养老金为主要目的的长期人身险，它是年金保险的一种特殊形式，又称为退休金保险，是社会养老保险的补充。

**I1.20.7** 其他养老保险，请注明________________

**I1.21** 目前，您是否有住房公积金？ 1.是 2.否 99999.不清楚

住房公积金是指国家机关、国有企业、城镇集体企业、外商投资企业、城镇私营企业及其他城镇企业、事业单位及其在职职工缴存的长期住房储金。

**I1.22** 目前，您是否有下列保险/福利？

**I1.22.1** 工伤保险 1.是 2.否 99999.不清楚

工伤保险是劳动者在工作中或在规定的特殊情况下，遭受意外伤害或患职业病导致暂时或永久丧失劳动能力以及死亡时，劳动者或其遗属从国家和社会获得物质帮助的一种社会保险制度。

**I1.22.2** 生育保险 1.是 2.否 99999.不清楚

生育保险是国家通过立法，在怀孕和分娩的妇女劳动者暂时中断劳动时，由国家和社会提供医疗服务、生育津贴和产假的一种社会保险制度，国家或社会对生育的职工给予必要的经济补偿和医疗保健的社会保险制度。

**I1.22.3** 失业保险 1.是 2.否 99999.不清楚

失业保险是指国家通过立法强制实行的，由社会集中建立基金，对因失业而暂时中断生活来源的劳动者提供物质帮助的制度。

**(二)教育经历**

**I2.1** 您的最高学历是（见编码3）：______（若在读，则以在读的最高学历为准）

访问员注意：未毕业算达到学历，如被访者读了小学，但小学未毕业，此题应该选“2.小学/私塾”；非全日制算达到学历，如被访者读的是非全日制本科，此题应选“8.本科”

1.未上过学 2.小学/私塾 3.初中

4.普通高中 5.职业高中 6.技校

7.中专 8.大专 9.本科

10.硕士 11.博士 99999.不清楚【跳至**I2.8**】

**I2.1.1** 您的最高学历是否毕业： 1已毕业 2仍在读 3肄业

【CAPI】 if I2.1.1==1 或3，则回答I2.1.2

**I2.1.2** 您的最高学历的结束年份是？________年

【CAPI】 if I2.1.1==2 或3，则回答I2.1.2.0

**I2.1.2.0** 您最高学历就读的年级是？____年级（比如本科三年级，此处填3，某人高中二年级肄业，此处填2）

【CAPI】 if I2.1==1，跳至I2.9； if I2.1==2，跳至I2.1.101；if I2.1==3，跳至I2.1.91

if I2.1==4，跳至I2.1.81； if I2.1==5，跳至I2.1.50 ； if I2.1==6，跳至I2.1.50

if I2.1==7，跳至I2.1.50 ； if I2.1==8，跳至I2.1.41 ； if I2.1==9，跳至I2.1.31

if I2.1==10，跳至I2.1.21； if I2.1==11，跳至I2.1.11

**I2.1.11**请问您的博士学历开始于哪一年？：_______年

**I2.1.12**您博士就读高等学校等级是（见编码6）（就读时学校等级）？

1.985高校 2.211高校 3.其他一本院校 4.二本院校 5.三本院校

1. 其他全日制高校（民办大学） 7.非全日制高校（函授，电大，刊大，夜大）

8.其他（请说明）_______

**I2.1.103.w16你的专业是________**

**I2.1.20**请问您是否读过硕士？ 1.是 2.否【跳至**I2.1.30**】

**I2.1.21** 请问您的硕士开始于哪一年？：_______年

**I2.1.22**请问您硕士就读高等学校等级是（见编码6）（就读时学校等级）？

1.985高校 2.211高校 3.其他一本院校 4.二本院校 5.三本院校

1. 其他全日制高校（民办大学） 7.非全日制高校（函授，电大，刊大，夜大）

8.其他（请说明）_______

**I2.1.104.w16你的专业是________**

**I2.1.30**请问您是否读过本科？ 1.是 2.否【跳至**I2.1.40**】

**I2.1.31** 请问您的本科开始于哪一年？：_______年

**I2.1.32**请问您本科就读高等学校等级是（见编码6）（就读时学校等级）？

1.985高校 2.211高校 3.其他一本院校 4.二本院校 5.三本院校

1. 其他全日制高校（民办大学） 7.非全日制高校（函授，电大，刊大，夜大）

8.其他（请说明）_______

**I2.1.105.w16你的专业是________**

**I2.1.40**请问您是否读过大专？ 1.是 2.否【跳至**I2.1.50**】

**I2.1.41** 请问您的大专开始于哪一年？：_______年

**I2.1.42**请问您大专就读高等学校等级是（就读时学校等级）？

1.专科一批 2.专科二批 3.非全日制高校（函授，电大，刊大，夜大）

4.其他（请说明）_______

**I2.1.106.w16你的专业是________**

**I2.1.50**请问您是否读过中专？ 1.是 2.否【跳至**I2.1.60**】

**I2.1.51** 请问您的中专开始于哪一年？：_______年

**I2.1.60**请问您是否读过职业高中？ 1.是 2.否【跳至**I2.1.70**】

**I2.1.61** 请问您的职业高中始于哪一年？：_______年

**I2.1.70**请问您是否读过技术学校？ 1.是 2.否【跳至**I2.1.80**】

**I2.1.71** 请问您的技术学校始于哪一年？：_______年

**I2.1.80**请问您是否读过高中？ 1.是 2.否【跳至**I2.1.91**】

**I2.1.81** 请问您的高中开始于哪一年？：_______年

**I2.1.82**请问您高中就读学校等级是（就读时学校等级）？

1.省/直辖市重点中学 2.县、市级重点中学 3.区、乡/镇重点中学

4.非重点中学 5.其他（请说明）_______ 6.不分重点非重点

**I2.1.91** 请问您的初中开始于哪一年？：_______年

**I2.1.92**请问您初中就读学校等级是（就读时学校等级）？

1.省/直辖市重点中学 2.县、市级重点中学 3.区、乡/镇重点中学

4.非重点中学 5.其他（请说明）_______ 6.不分重点非重点

**I2.1.101** 请问您的小学/私塾开始于哪一年？：_______年

**I2.8** 请回忆一下您14岁还在学校读书时 (大概是初二/初三)的情况,【如果那时您已经不读书，那就请回忆更早您还没有离开学校时】。您同不同意下面几点对您那时情况的说法？

| 情况 | 1.非常不同意 | 2.不同意 | 3.同意 | 4.非常同意 | 5.不适用 |
| --- | --- | --- | --- | --- | --- |
| I2.8.1 就算身体有点不舒服，或者有其他理由可以留在家里，我仍然会尽量去上学. |  |  |  |  |  |
| I2.8.2就算是我不喜欢的功课，我也会尽全力去做。 |  |  |  |  |  |
| I2.8.3就算功课需要花好长时间才能做完，我仍然会不断地尽力去做。 |  |  |  |  |  |

**I2.9** 2015年7月以来您是否有参加过至少5天的专业技术培训？ 1.有 2.没有

**I2.10** 您是否获得过专业技术资格证书？ 1.有； 2.无**（**跳答I2.12**）**

**I2.11** 您获得过的专业技术资格证书一共有多少个？_____个（答案为0则跳至I2.12）

I2.11.1 如果获得的资格证书超过三项，请填写被访者认为最重要的三个（见编码7）

|  | 获得证书时间 | 专业资格证书名称 | 是否与你的工作相匹配  1是 2否 |
| --- | --- | --- | --- |
| 1 | ______年 |  |  |
| 2 | ______年 |  |  |
| 3 | ______年 |  |  |

证书名称编码：

1、法律类 2、管理、咨询、商务、市场营销类 3、经济专业技术、评估、拍卖类

4、房地产、金融、保险类 5、统计、会计、税务、审计类 6、语言、教育、出版类

7、计算机应用及软件类（IT类） 8、建筑工程、城市规划类 9、医务、药业类

10、其他_______

**I2.12**请您评估一下您在以下方面的能力？

|  | 0完全没问题 | 1还可以 | 2不太行 | 3完全不会 |
| --- | --- | --- | --- | --- |
| 阅读报刊 |  |  |  |  |
| 写信 |  |  |  |  |
| 用手机发短信 |  |  |  |  |
| 使用网上银行 |  |  |  |  |
| 网上购买火车票 |  |  |  |  |
| 银行ATM取款 |  |  |  |  |

**I2.14** 您的出生地：_____**省**（自治区、直辖市）、____**市**（地、盟、州）、___**县**(区、旗、县级市)

**I2.15** 您14岁的居住地跟你的出生地是一样的吗？ 1.是（跳至I2.16.0 ） 2.否

**【data】 如果I2.15==1，生成I2.16==I2.14**

**I2.16** 您14岁的居住地是在哪里？

_____**省**（自治区、直辖市）、____**市**（地、盟、州）、___**县**(区、旗、县级市)

**I2.16.0** 请问您自14岁以来，您是否有过跨县市迁移经历？ 1.是（跳至**I2.16.2** ） 2.否

（迁移经历指从一个县市迁移到另一个县市，并连续居住**六个月**及以上；同一个城市内区与区之间的迁移不算，同一城市内县与区之间的迁移算；如果被访者目前到本地居住不满半年，也算一次迁移经历)

【CAPI】 if I2.16 的县区编码与现居住地的县区编码不一样，则询问I2.16.1

I2.16.1 您14岁的居住地与现居住地不一样，请您确认有没有迁移过？ 1.迁移过 2.没有迁移过（跳至I3.0）

（请访问员根据迁移的定义，向被访者进行确认，如果确实为迁移过的，请选择是）

**I2.16.2** 您14岁以来，第一次迁移发生在哪一年？______年

**I2.16.3**您14岁以来，第一次迁移是迁移到什么地方的？___省___市/县

**I2.16.4** 您14岁以来，第一次迁移的迁移原因是（多选，选项见编码5）

1.务工经商 2.工作调动 3.学习培训

4.分配录用 5.家属随迁 6.拆迁搬家

7.婚姻 8.转干 9.参军

1. 支内/支边 11.上山下乡 12.其他（请注明）_____

I2.16.4.1.w16 请问您迁移到这个地方后的首份职业是什么_______

1. 国家机关、党群组织、企业、事业单位负责人 2.专业技术人员 3. 办事人员和有关人员、4. 商业、服务业人员 5. 农、林、牧、渔、水利业生产人员6. 生产、运输设备操作人员及有关人员 7. 不便分类的其他从业人员 8.迁移到此地后未工作过

I2.16.5 除了这一次迁移，您之后还有迁移过吗？ 1.有 2.没有

【CAPI】 if I2.16.3 的县区编码与现居住地的县区编码不一样，则询问I2.16.6

I2.16.6 您这一次的流入地与现居住地不一样，请您确认有没有迁移过？ 1.迁移过 2.没有迁移过（跳至I3.0）

（迁移经历指从一个县市迁移到另一个县市，并连续居住六个月及以上；同一个城市内区与区之间的迁移不算，同一城市内县与区之间的迁移算；如果被访者目前到本地居住不满半年，也算一次迁移经历；)

**I2.17.2** 您14岁以来，第二次迁移发生在哪一年？______年

**I2.17.3**您14岁以来，第二次迁移是迁移到什么地方的？___省___市/县

**I2.17.4** 您14岁以来，第二次迁移的迁移原因是（多选，选项见编码5）

1.务工经商 2.工作调动 3.学习培训

4.分配录用 5.家属随迁 6.拆迁搬家

7.婚姻 8.转干 9.参军

1. 支内/支边 11.上山下乡 12.其他（请注明）_____

I2.16.4.1.w16 请问您迁移到这个地方后的首份职业是什么_______（）

1. 国家机关、党群组织、企业、事业单位负责人 2.专业技术人员 3. 办事人员和有关人员、4. 商业、服务业人员 5. 农、林、牧、渔、水利业生产人员6. 生产、运输设备操作人员及有关人员 7. 不便分类的其他从业人员 8.迁移到此地后未工作过

I2.17.5 除了这一次迁移，您之后还有迁移过吗？ 1.有 2.没有

【CAPI】 if I2.17.3 的县区编码与现居住地的县区编码不一样，则询问I2.17.6

I2.17.6 您这一次的流入地与现居住地不一样，请您确认有没有迁移过？ 1.有迁移过 2.没有迁移过（跳至I3.0）

（请访问员根据迁移的定义，向被访者进行确认，如果确实为迁移过的，请选择是）

【CAPI】按照此规则一直往下询问，知道访问员与被访者确认没有迁移过为止。

**（三）工作状况**

**I3.0** 您是否有工作经历？

**访问员注意**：必须将下段话读出“工作指从事有收入的活动，**务农**、兼职、帮忙家庭生意也算在内，但不包括义工、学生兼职、志愿者、家务劳动这些活动”

1.是(跳至**I3.4.w16** ) 2.否

**I3.0.1**我们说的工作**指从事有收入的活动**，务农、兼职、帮忙家庭生意也算，但不包括义工、学生兼职、志愿者、家务劳动这些活动，请您确定自己有没有过工作经历？

1.有过工作经历 2.没有过工作经历**（返回I3.0修改）**

**【data】生成是否有过工作经历的变量work**

**I3.4.w16** 上周你是否做过家务（如做饭、洗碗、洗衣服、打扫房间、带小孩等）？

      1.是      2.否（跳答I3.1）

**I3.4.1.w16** 家务劳动平均每天花费您多长时间？________分钟

【CAPI】if work=1 则继续询问；if work=2，则跳至I3c.4

**I3.1** 2015年1月以来，您是否工作过？

| 1.工作过（工作指从事**有收入的活动**，务农、兼职、帮忙家庭生意也算在内，不包括义工、学生兼职、志愿者、家务劳动这些活动）(跳至I3.2) | |
| --- | --- |
| 2.没有工作过 |  |

【data】if I3.1==1则生成2015年1月以来是否工作过的变量 work2015==1

**I3.1.1**现在有工作也表示2015年1月份以来工作过，2015年1月份以来务过农也表示工作过，您确定2015年 1月以来，您没工作过？

1.工作过 2.没有工作过 **（跳至C）**

【data】if I3.1.1==1，则recode work2015(.==1)

**I3.2** 您是否有农业生产经历（指务农这一职业，不包括还是学生的时候帮忙干农活的情况）？

1.有 2.否**（跳至I3a.1）**

**I3.3** 您有多长农业生产经历？_______年

1. **过去一年有工作者**

**I3a.25.w16** 您目前或最近这份工作是什么时候开始的？_______年

**I3a.1** 您目前或最近一份工作一般一周工作几小时？_______小时

**I3a.2** 您过去一周工作几小时？_______小时

**I3a.3** 您目前或最近一份工作一般一个月工作几天？_______天

**I3a.4** 您过去一个月工作几天？_______天

**I3a.5** 您过去一年工作几个月？_______月（访问员注意：工作不足一个月按一个月计算）

**I3a.5.0** 请问您工作的地方主要在哪里？

1. 本村居 2. 本乡镇的其他村居 3. 县/区内的其他乡镇(不含县/区城)

4. 县/区城 5. 本县/区以外

**I3a.5.1**您目前或最近的工作，每天上班和下班在路途上要花费的时间一共是______小时_____分钟

**I3a.5.2** 您上下班/外出劳动的主要方式是（多选）

1. 走路 2.自行车 3.摩托车/电动车 4.公交车/班车

5.地铁 6.出租车 7.私家车 8.基本呆在家里

**I3a.5.3**您目前或最近的工作，工作日一般每天上班和下班所花的路费一共是_______元

**I3a.6** 您2015年各类收入总计是（包括农业收入，工资收入，经营收入等，自给自足的农业生产需按市场价值折算成收入）**:____元**

**I3a.6.1** 其中，您2015年的扣除个人所得税,社会保险，住房公积金后的工资性收入（包括所有的工资、各种奖金、补贴，）：_____元（工作不足一年，则年工薪收入为平均每个月收入×12）

**I3a.6.2**不扣除个人所得税，社会保险和住房公积金，您2015年的工资性收入有多少？_ __元

【CAPI】 if I3a.6.1不等于99997 99998 99999

且I3a.6.2不等于99997 99998 99999

且I3a.6.2<=I3a.6.1，则询问I3a.6.3，否则跳至I3a.6.4

**I3a.6.3**不扣除个人所得税，社会保险和住房公积金，您2015年的工资性收入确定是有多少？_ __元

**I3a.6.4** 其中，您2015年经营性收入（经营性收入指的是农业经营收入，自给自足的农业生产需按市场价值折算成收入，店铺，工厂,生意经营收入，这里记录的是税后纯收入）：________元

**I3a.7.0** 您现在的工作情况属于以下哪种？

1.有工作 2.无工作

访问员注意：工作指从事有收入的活动，务农、兼职、帮忙家庭生意也算在内，不包括义工、学生兼职、志愿者、家务劳动这些活动。

【CAPI】

#1如果I3a.7.0=1则**I3a.7**询问“您目前工作”工作情况

#2如果I3a.7.0=2则**I3a.7**询问“您2015年1月份以来的最近这份工作”的工作情况

**I3a.7** 您目前或2015年1月份以来的最近一份工作是**________**（请填具体工作内容）

访问员注意：请填具体工作内容，若被访者有多份工作则填写最主要的那份工作

**I3a.8** 您*I3a.7*的工作属于什么行业类型：_____种类(看编码)

**I3a.9** 您* I3a.7*这份工作单位属于以下哪种（见编码8）：_________

（访问员注意：单位应该是一个独立的机构，有自己的财务和人事管理职权。如果受访者的工作机构分很多层级，无法区分哪一级是自己的单位时，可以提示，受访者工资关系所在的那一级，就可能是他/她的单位：由劳务派遣机构派出的保安，劳务工，家政服务员等，劳务派遣机构是其单位；个体经营者也要填答。如果被访者的工作没有单位，则填答10或11.）

【有单位】

1.党政机关、人民团体、军队 2.国有/集体事业单位 3.国营企业

4.集体企业 5.村居委会等自治组织 6.民营、私营企业

7.外资、合资企业 8 民办非企业、社团等社会组织

9.个体工商户（包括登记过的个体工商户或未登记的各类店主）

【无单位】

10.务农：农林牧副渔业生产（如种地、养殖鸡鸭水产等）

11.自由工作者（自由职业者，零散工，摊贩，无派遣单位的保姆，自营运司机，手工工匠等）

【CAPI】如果I3a.9 =1或2或3或4，询问I3a.9.1.w16

I3a.9.1.w16你是否有编制/你是否在编制内？

1是 2否

I3a.20.w16在您的工作中，以下的事情在多大程度上由自己来决定的？

|  | 1.完全由  自己决定 | 2.部分由  自己决定 | 3.完全由  他人决定 |
| --- | --- | --- | --- |
| 1．工作任务的内容 | 1 | 2 | 3 |
| 2．工作进度的安排 | 1 | 2 | 3 |
| 3．工作量/工作强度 | 1 | 2 | 3 |

I3a.21.w16您的居住地或工作地周边近几年是否有企业迁入（不包括新建的企业）？

1. 有 2. 没有 3.不清楚

I3a.22.w16您的居住地或工作地周边近几年是否有企业迁出？

1.有 2. 没有 3不清楚

【CAPI】if I3a.9= 6 7 8 9，则询问I3a.10,否则跳至I3a.13

**I3a.10** 您是您当前所在单位的老板（包括合伙人，所有者，法人代表等）还是雇员？

1老板（包括合伙人，所有者，法人代表等） 2雇员

【CAPI】if I3a.10==1则询问：

**I3a.11** 您* I3a.7*的工作机构登记的执照类型是？

1无执照 2个体户 3私营企业 4民办非企业，社团，基金会

5.其他（请注明）______

**I3a.11.1**您从申请到拿到营业执照经历了________天

【CAPI】if I3a.11不等于3，则询问：

**I3a.12**您* I3a.7*的工作有没有雇佣其他人？（雇佣他人指的是给他人发工资）

1是 2否

**I3a.13**您* I3a.7*的工作是否有固定的工作场所？ （）

1.是 2.否

**I3a.14**您* I3a.7*的工作的工作场所主要是：

1. 户外 2. 车间 3. 室内营业场所 4. 办公室

5. 家里 6. 运输工具内 9. 其他【请注明】____

**I3a.15**您* I3a.7*的工作是否是一份全职工作？（每周工作超过35小时，即每天工作7小时，每周工作5天） 1.是 2.否

【DAIA】

如果I3a.9==10时，判定被访者职业类型为务农，生成I3a.16==4

如果I3a.9=1 2 3 4 5或I3a.10 =2时，判定该被访者职业类型为雇员，生成I3a.16==1

如果I3a.11=3或者I3a.12=1时，判断被访者职业类型为雇主，生成I3a.16==2

如果I3a.9=11或I3a.12==2时，判断被访者的职业类型为自雇，生成I3a.16==3

**I3a.17** 如果将工作区分为雇员，雇主，自雇和务农四种，您看看下面的定义，我们认为您* I3a.7*这份工作应该属于* I3a.16*，您认为正确吗？（访问员必须和被访者一起进行解释和确认）

1.正确 2不正确

1雇员：受雇于他人或组织，政府部门以及国有企业，事业单位所有员工都算受雇于国家的雇员

2雇主：雇佣1人及以上的个人，雇佣指的是给人发工资

3自雇：其工作的雇主就是自己，同时也不雇佣他人工作，他们不隶属于任何组织

4务农：指从事农业生产活动人员

**I3a.18 正确的工作状态应该是**

1雇员 2雇主 3自雇 4务农

【DAIA】 如果I3a.17=2,生成I3a.16= I3a.18

**I3a.19 修改的原因是_____________**

例：之前单位类型答错，应该是个体户

被访者不是雇员，是和丈夫合作开店的，应该算合伙人

被访者有稳定的雇主，应该算雇员而不是自雇

【CAPI】if I3a.7.0=2则跳至I3c.1

3# if I3a.7.0=1且I3a.16==3，则直接跳至I3a3.1

4# if I3a.7.0=1且I3a.16==4，则直接跳至I3a5.1

I3a.23.w16 您所在的工作单位，近几年有发生过跨县区的部分或全部搬迁吗？

1.有 2.没有

【CAPI】如果I3a.23.w16=1，则继续回答I3a.23.1.w16，否则跳至I3a.24.w16

I3a.23.1.w16 搬迁的方向是怎样的？

1.从边缘向中心、或从经济不发达地区向发达地区搬迁

2.从中心向边缘、或从发达地区向不发达地区搬迁

3.和原来的差不多

I3a.23.2.w16 搬迁的范围是怎样的？

1.同一城市/地区内的搬迁

2.同一省份的不同地区/城市的搬迁

3.不同省份的搬迁

I3a.23.3.w16 据您估计，您所在单位的迁移对您在被迁移部门的同事的以下方面有怎样的影响？

|  | 显著变坏 | 轻微变坏 | 一无影响 | 轻微变好 | 显著变好 | 不清楚 |
| --- | --- | --- | --- | --- | --- | --- |
| 收入 |  |  |  |  |  |  |
| 生活环境 |  |  |  |  |  |  |
| 发展机会 |  |  |  |  |  |  |

I3a.24.w16 您所在单位未来几年是否有部分或全部迁移的打算？

1.有（继续回答） 2.没有（跳答）

【CAPI】

1# if I3a.24.w16=1，则继续回答I3a.24.1.w16；

2# if I3a.7.0=1且I3a.16==1且I3a.24.w16=2，则跳至I3a1.1；

3# if I3a.7.0=1且I3a.16==2且I3a.24.w16=2，则跳至I3a2.1

I3a.24.1.w16 搬迁的方向是怎样的？

1.从边缘向中心、或从经济不发达地区向发达地区搬迁

2.从中心向边缘、或从发达地区向不发达地区搬迁

3.和原来的差不多

I3a.24.2.w16 搬迁的范围是怎样的？

1.同一城市/地区内的搬迁

2.同一省份的不同地区/城市的搬迁

3.不同省份的搬迁

I3a.24.3.w16 您是否有跟随单位搬迁的打算？

1. 有 2. 没有

【CAPI】

1# if I3a.16==1，则跳至I3a1.1

2# if I3a.16==2，则跳至I3a2.1

**A1、雇员**

【CAPI】 if I3a.16=1 则需要回答A1雇员部分的问题

**I3a1.1** 您是否有固定的雇主？

1.是 2.否

**I3a1.2** 您现在的工作是否是为您自己的家庭/家族的企业/公司/生意做的？

1.是 2.否

**I3a1.3.1** 你目前工作的单位/企业包吃吗？ 1是 2否(跳至**I3a1.3.2**)

**I3a1.3.1.1**每月能为你节省多少钱？_______元/月

**I3a1.3.2**您目前工作的单位/企业包住吗？ 1是 2否(跳至**I3a1.4**)

**I3a1.3.2.1**每月能为你节省多少钱？_______元/月

**I3a1.4** 您目前工作的工资计算方式是：

1.计件 2.计时 3.按天计算

4.有时计件，有时计时 5.月薪制 6.提成或底薪加提成

7.绩效工资或底薪加绩效工资 8.年薪制 9.其他(请注明)_______

**I3a1.5** 目前是否签订书面劳动合同？

1.是 2.否**(跳答I3a1.9)**

**I3a1.6** 本次合同是哪年签订的？__________年

**I3a1.6.1**您是否保存了合同的原件或复印本？ 1.是 2.否

**I3a1.7** 您现在的劳动合同是与谁签订的？

1.工作所在的公司（单位） 2.某中介劳务公司（劳务派遣）

**I3a1.8** 您签订的是哪种类型的劳动合同？

1.固定时段或短期合同 2.永久合同**（跳至I3a1.9）**

**I3a1.8.1** 这是您第几次与本单位/企业签订合同？________次

**I3a1.8.2** 这次合同，您与本单位/企业签订了几年？__________年

**I3a1.9**在您看来，要做好这个工作，是否需要接受专门的训练或培训？

1.需要 2.不需要

**I3a1.10** 为掌握这份工作所需要的主要技能，您花了多少时间？（单选）

1.一天 2.几天 3.大约一周 4.不到一个月

5.一个月到三个月 6.超过三个月，不到一年 7.一年以上 8.三年以上

【CAPI】 if性别=女 有婚姻经历，年龄<52，填答I3a1.13，否则跳至I3a1.14

**I3a1.13 （注意：只问所有52岁以下妇女）**2015年7月份以来您在该工作单位工作期间是否有过生育经历？

1.是 2.否**(跳至I3a1.14)**

**I3a1.13.1** 您最近一次的产假是多久？___________月；

**I3a1.13.2**您这次产假是否是带薪休假？ 1.是 2.否

**I3a1.13.3**产假结束回来工后，工作单位是否因您休了产假而对您有降薪，降职等行为？

1.有 2.没有

**I3a1.14.0** 通常情况下，您是否加班？

1.是 2.否（跳至I3a1.14 ）

**I3a1.14.1** 通常情况下，您的加班符合下列哪种情况？

1.工作日延时加班 2.休息日加班 3.法定节假日加班

**I3a1.14.2** 通常情况下，您加班的地点是？

1.工作地点 2.工作场所以外的其他地点（如宿舍、家、酒店等）

**I3a1.14.3.** 通常情况下，您在目前工作单位的加班是自愿的吗？

1.是 （跳答I3a1.14.4） 2.不是 （跳答I3a1.14.5）

**I3a1.14.4.** 通常情况下，您自愿加班的主要原因是：

1.为增加收入；

2.为获得升迁的机会；

3.为企业分忧；

4.为证明自己的工作能力

5.其他(请注明)_________________。

**【CAPI】**I3a1.14.4回答完毕，跳至I3a1.14.6

**I3a1.14.5.** 通常情况下，您非自愿加班的主要原因是

1. 单位规定必须加班，不加班会受罚，甚至失去工作（加班作为一种明文规定）
2. 加班被列入工作表现的考核范围，涉及晋升机会 （加班作为一种“潜规则”）

3、大家都加班，我也只好加班（加班作为一种“职场亚文化”）

4、其他(请注明)：_______

**I3a1.14.6** 通常情况下，您的加班补偿类型符合下列哪种？

1. 加班费或奖金 2. 补休或调休 3. 非实质性补偿如绩效评分或升迁机会

4.其他补偿 5.无任何补偿

**I3a1.14** 过去一个月，您是否有加班过？

1.是 2.否（跳至I3a1.15）

**I3a1.14.7** 您上个月共加班多少小时？_______小时，其中有报酬的有_______小时

**（无任何补偿则为0小时）**

**I3a1.14.8** 您上个月获得的加班工资是多少__________元？

（**无任何补偿则为0元**）

**I3a1.15** 您是否有直接的下属？

1.有 2.没有 **（**跳至I3a1.16**）**  99998.不适用**（**跳至I3a1.16**）**

**I3a1.15.1** 您的直接下属有无直接下属？

1.有 2.没有 99998.不适用

**I3a1.15.2** 您的下属共有多少人？________人

**I3a1.17** 您的单位/企业现在有没有工会？

1.有； 2.没有**（跳答I3a1.18）**  99999.不清楚**（跳答I3a1.18）**

**I3a1.17.1** 您是否是加入了工会？

1.是，主动加入的 2.是，被动加入的

3.是，自动成为会员 4.没加入 99999.不清楚

**I3a1.17.2** 去年您是否参加单位/企业的工会活动？ 1.是 2.否

**I3a1.17.3** 您单位/企业的工会对您有帮助吗？ 1.有 2.无

**I3a1.18** 自2015年7月份以来，您是否遇到过下列问题?

| 问题 | 1.有  2.否  **（跳至下一项）** | 您采取了哪些行动（多选）？（按时间顺序回答）（代码1，见编码9） | 问题解决了吗？  1.部分解决  2.全部解决  3.正在处理中**（跳至下一项）**  4. 未解决**（跳至下一项）** | 您认为使得这个问题得以解决的最重要  的行动是什么？  【CAPI】选项为  选择的采取了的行动 |
| --- | --- | --- | --- | --- |
| **I3a1.18.1**、拖欠工资 |  |  |  |  |
| **I3a1.18.2**、强制加班 |  |  |  |  |
| **I3a1.18.3**、工伤 |  |  |  |  |
| **I3a1.18.4**、工作的安全保护未达到国家标准 |  |  |  |  |
| **I3a1.18.5**、工作环境污染超过标准 |  |  |  |  |
| **I3a1.18.6**、正式要求增加工资 |  |  |  |  |
| **I3a1.18.7**、要求雇主为你缴纳社会保险（如养老，医疗等） |  |  |  |  |

**代码1：** 1.集体与本单位协商 2.个人与本单位协商

3.找工会 4.找法院

5.找媒体 6.集体行动（例如上访、罢工、散步等）

7.个体抗争（停工、上访等） 8.辞职

9.找劳动争议仲裁委员会 10.找其他组织或社会团体

11.上网反映情况 12.没有采取任何行动

**I3a1.18.8**您工作的单位是否有关于罚款的制度？ 1.有 2.没有（跳至**I3a1.34.w16**）

**I3a1.18.9**过去一个月，您在该单位是否被罚款过？ 1.是（金额是_________元） 2.否

**I3a1.34.w16**如果您的劳动权益受到侵犯，对于以下几种途径，您参加或采用的意愿如何？

|  | 非常愿意 | 比较愿意 | 看情况 | 不大愿意 | 非常不愿意 | 不清楚 | 拒绝回答 |
| --- | --- | --- | --- | --- | --- | --- | --- |
| **I3a1.34.1.w16**静坐、堵马路 | 1 | 2 | 3 | 4 | 5 | 99998 | 99999 |
| **I3a1.34.2.w16**罢工 | 1 | 2 | 3 | 4 | 5 | 99998 | 99999 |
| **I3a1.34.3.w16**上访 | 1 | 2 | 3 | 4 | 5 | 99998 | 99999 |
| **I3a1.34.4.w16**找劳动部门 | 1 | 2 | 3 | 4 | 5 | 99998 | 99999 |
| **I3a1.34.5.w16**找老乡帮忙 | 1 | 2 | 3 | 4 | 5 | 99998 | 99999 |
| **I3a1.34.6.w16**找律师帮忙 | 1 | 2 | 3 | 4 | 5 | 99998 | 99999 |

**I3a1.19** 您在工作过程中，是否需要（单选）：

|  | 经常 | 有时 | 很少 | 从不 |
| --- | --- | --- | --- | --- |
| **I3a1.19.1** 繁重的体力劳动 | 1 | 2. | 3 | 4. |
| **I3a1.19.2** 频繁地移动身体的位置 | 1 | 2. | 3 | 4. |
| **I3a1.19.3** 快速反应的思考或脑力劳动 | 1 | 2. | 3 | 4. |
| **I3a1.19.4**使用互联网 | 1 | 2. | 3 | 4. |

**I3a1.25** 您上周有没有去工作？ 1.有（跳至3B兼业部分） 2.没有

（访问员注意：在家办公不算没有去工作）

**I3a1.26** 到上周周末为止，您不工作多长时间？

**I3a1.26.1**如果是几周，那么有________周

**I3a1.26.2**如果是几个月，那么有______个月

**I3a1.26.3**如果是几年，那么有________年

**I3a1.27** 上周，您没有做您的工作的主要原因是（见编码10）？

1.由于计划改变、原材料短缺、没有订单等暂时不能开工

2.工作时间和要求比较宽松 3.等着开始一份新的工作

4.因劳动纠纷而引起的罢工等 5.为了照顾小孩

6.为了照顾家中其他人 7.天气原因影响工作

8.休产假/待产 9.伤病

10.上学/培训 11.正常休假（调休或寒暑假）

12.其他（请说明）________

**I3a1.28** 上周，您没有去工作是不是也有工资？ 1.是 2.否

**I3a1.29** 您的雇主是否给了一个明确的时间让您回去工作？ 1.是 2.否

**I3a1.30** 您是否听到一些您将在半年内返回那份工作的消息？ 1.是 2.否

**I3a1.31** 如果上周您被通知继续去工作，您去得了吗？ 1.是 2.否

**I3a1.32** 如果您不能返回工作，原因是？

1.仍然伤病 2.仍然照顾小孩

3.仍然照顾家中其他人 4.仍然休产假/待产

5.上学 6.其他(请说明）_______

**I3a1.33** 在过去的4周内您是否找了其他工作？

1.是 2.否

（答完跳至3B兼业部分）

**A2、雇主**

**I3a2.1** 您是什么开始现在这份生意/经营的？______________年

在创业初期是否是兼职创业（即在从事固定职业同时创业）？ 1是 2否

**I3a2.1.1**您现在这份生意/经营的工作内容是什么？________

范例：开小店卖服装；开工厂生产毛毯；开酒店，旅馆等

**I3a2.2** 您现在这份生意/经营，开始时注册登记形式是？

1.个体户 2.私营企业

3.民办非企业 4.其他（请注明）：______

**I3a2.3** 您现在这份生意/经营，开业时投入资金大约是________万元

**I3a2.3.1**开业时投入资金的来源比例？（多选改为填空）

1.个人积累比例_______ 2.家人和亲属资助比例_____

3.朋友、生意伙伴与其他社会关系比例____ 4.政府和其他金融机构投入比例____

**I3a2.4** 您个人现在在该生意上的资产总额有__________万元；负债________万元（如果有多个生意项目，请填答主要生意的资产总额）

**I3a2.31.w16**请问您企业近三年的营业收入和利润状况如何？请填写具体数值。

营业收入：2013年________ 万元； 2014年________ 万元； 2015年________ 万元。

净 利 润：2013年________ 万元； 2014年________ 万元； 2015年________ 万元。

**I3a2.5** 产权性质： 1.独资**（跳至I3a2.8）**  2.合资

**I3a2.6** 您生意目前共有几个企业(法人）投资者？ 位

**I3a2.6.1** 您生意目前共有几位个人（自然人）投资者？ 位

**I3a2.6.2** 您或您的小家庭（包括夫妻和子女）所占有的比例（份额）：_________%，亲属（父母兄弟等）的比重 %

**I3a2.7您与目前合创业合伙人之间的关系(多选)：**

1.家人（包括夫妻、父母子女和兄弟姐妹） 2.其他亲属

3.朋友（其他社会关系） 4.单纯商业合作关系 5.其他_______

**I3a2.32.w16**您与合作者之间是否签署了正式的投资协议？ 1.是 0.否

**I3a2.8**您这次创业是因为找到好的创业机会还是因为您没有更好的工作选择？

【读出】

1.抓住好的创业机会 2.没有更好的工作选择

【不读出】

3.以上两个都是 4.当时有好的工作岗位，但创业机会更加好

【CAPI】 if I3a2.8==1或I3a2.8==3或I3a2.8==4, 则询问I3a2.8.1

**I3a2.8.1** 您的创业机会来自于下列哪种渠道？（选项修改）

1.原来的工作经历 2.自己的发现

3.朋友的介绍 4.对海外企业的模仿和借鉴（？）

5.模仿身边的其他企业 6其他，请注明.

**I3a2.33.w16**您认为以下因素对您创业的重要程度如何？请在最能代表您真实意见的数字上打勾。

|  | 非常不重要 | 不重要 | 一般 | 重要 | 非常重要 |
| --- | --- | --- | --- | --- | --- |
| 有技术（如：有相关研究成果或专利） | 1 | 2 | 3 | 4 | 5 |
| 有经营经验 | 1 | 2 | 3 | 4 | 5 |
| 有人脉关系支持 | 1 | 2 | 3 | 4 | 5 |
| 有经济基础 | 1 | 2 | 3 | 4 | 5 |
| 有政府支持 | 1 | 2 | 3 | 4 | 5 |

**I3a2.9** 您第一年所做的生意（订单）来自下列渠道的比例：

1.党政机关和国有企事业单位 ______ 2.集体企业______

3.其他私营企业和个体户______ 4.外资/合资企业______

5.最终消费者______

**I3a2.18** 过去一年以来您的生意竞争激烈吗？

1.非常激烈 2.比较激烈 3.不太激烈 4.根本不激烈 5.没有竞争

9.不好说

**I3a2.10** 生意第一年竞争激烈吗？

1.非常激烈 2.比较激烈 3.不太激烈 4.根本不激烈 5.没有竞争

9.不好说

**I3a2.12** 您生意开始时，有多少人主动给您提供生意项目？人数是_____

【CAPI】 if I3a2.12=0或I3a2.12=99998，则跳至I3a2.14

**I3a2.13** 您与提供生意的人以前认识吗?（提供最重要的生意项目的那位）

1.认识 0.不认识**(**跳至I3a2.14**)**

**I3a2.34.w16**您与他/她是何种关系？

1.家人（包括夫妻、父母子女和兄弟姐妹） 2.其他亲属

3.朋友（其他社会关系） 4.单纯商业合作关系 5.其他_______

**I3a2.34.1.w16**他/她在你的生意中是否入股？

1.是 0.否

**I3a2.13.5**您们的信任程度是:

1.信任极了 2.很信任 3.较信任 4.不太信任 5.不信任

**I3a2.14**过去一年，您有多少生意来自于自己和合作者的人脉关系（按销售额的比例）？  **%【填答0跳至I3a2.18】**

**I3a2.15**介绍最多生意的人来自下列哪个类别？( 见编码15）

1.家人（包括夫妻、父母子女和兄弟姐妹） 2其他亲属

3.熟人和朋友 4.工商联或其他协会商会人士

5中介机构 6.其他_______

7.没有人介绍 **(跳至I3a2.18)**

**I3a2.35.w16**你认为和下面哪些单位打好关系对你的生意是重要的? (多选)

1.党政机关 2.国有企业 3.国有事业 4.集体企业

5.个体经营 6.私营企业 7.外资/合资 8.股份制企业

9.工商联 10.企业家协会 11.行业协会 12.专业协会

【CAPI】if I3a2.1>2015 则跳过I3a2.21，跳至I3a2.22

**I3a2.21**过去一年中，每个月您在生意上与政府打交道的平均次数是______次？

**I3a2.21.1.w16**过去一年您招待政府官员的开支大约______元？

**I3a2.21.2.w16**招待客户的开支大约______元？

【CAPI】 if I3a.11==3且I3a.12==2，则询问I3a2.22,否则跳至I3a2.23

**I3a2.22**  您现在为什么没有雇人？

1季节性用工，现在不需要雇人 2.暂时歇业 3.企业关门 4.其他（请注明）___

**I3a2.23** 您目前的生意雇员人数（雇员是指给他发工资）：_______人，

其中亲属（含血缘亲属和婚姻亲属）：______人

您的子女人数：______人

**I3a2.24** 雇员性别：男_____人；女______人

**I3a2.25** 雇佣方式（多选）

1.文字合同 2.口头协议 3.其他（请说明）__________

**I3a2.26** 过去一个月，您雇佣的雇员的平均工作时间：______天

**I3a2.27** 过去一个月，您雇佣的雇员平均每周加班时间：______小时

**I3a2.28**过去一个月，您所雇佣的员工的最高工资是__________元；您所雇佣的员工的最低工资__________元；总工资________元

**I3a2.29** 2015年，您生意的用工成本占总产值比例为： __________%

**I3a2.30** 2015年，您生意缴纳的税费占生意收入的比例为： __________%

**I3a2.30 .1.w16** 2015年缴纳的行政事业性收费占生意收入的比例为__________%，

**I3a2.30 .2.w16** 2015年税后纯利润中用于再投资的比例为__________%

**I3a2.36.w16** 您与政府部门打交道过程中，是否会寻求身边亲人朋友提供帮助？

1.总是 2.经常 3.一般 4.较少 5.从不

**I3a2.37.w16** 请您对贵企业所在省的制度环境进行评价。

|  | 非常不同意 | 不同意 | 不同意也不反对 | 同意 | 非常同意 |
| --- | --- | --- | --- | --- | --- |
| 现有的法律法规很完善 | 1 | 2. | 3 | 4. | 5. |
| 私有产权能得到很好的保护 | 1 | 2. | 3 | 4. | 5. |
| 政府以及相关部门积极鼓励创业 | 1 | 2. | 3 | 4. | 5. |
| 各级政府对创业都有特殊的优惠政策 | 1 | 2. | 3 | 4. | 5. |
| 各级政府对创业有各种各样的资助 | 1 | 2. | 3 | 4. | 5. |
| 各级政府对创业失败的企业也会有各种各样的帮助 | 1 | 2. | 3 | 4. | 5. |
| 各级政府的政府采购合同会优先考虑有创业项目的企业 | 1 | 2. | 3 | 4. | 5. |

**(访问员注意：答完A2，**跳至3B兼业部分）

**A3、自雇**

**I3a3.1**您这次创业是因为找到好的创业机会还是因为您没有更好的工作选择？

99998.**不适用**，因为被访者是建筑工，零散工，保姆及其他非创业的情况（跳至I3a3.3）

【读出】

1.抓住好的创业机会 2.没有更好的工作选择

【不读出】

1. 以上两个都是 4.当时有好的工作岗位，但创业机会更加好

【CAPI】 if **I3a3.1**==1或**I3a3.1**==3或**I3a3.1**==4, 则询问I3a3.2.1.w16，否则跳至I3a3.2.2.w16

**I3a3.2.1.w16** 您的创业机会来自于下列哪种渠道？（选项修改）

1.原来的工作经历 2.自己的发现

3.朋友的介绍 4.对海外企业的模仿和借鉴

5.模仿身边的其他企业 6其他，请注明.

**I3a3.2.2.w16**您认为以下因素对您创业的重要程度如何？请在最能代表您真实意见的数字上打勾。

|  | 非常不重要 | 不重要 | 一般 | 重要 | 非常重要 |
| --- | --- | --- | --- | --- | --- |
| 有技术（如：有相关研究成果或专利） | 1 | 2 | 3 | 4 | 5 |
| 有经营经验 | 1 | 2 | 3 | 4 | 5 |
| 有人脉关系支持 | 1 | 2 | 3 | 4 | 5 |
| 有经济基础 | 1 | 2 | 3 | 4 | 5 |
| 有政府支持 | 1 | 2 | 3 | 4 | 5 |

**I3a3.3** 您目前这份工作有没有固定的工作时间？

1.无固定工作时间 2.有固定工作时间**（跳至I3a3.5）**

**I3a3.4** 您一般一天的工作时间最长是____小时；最短是____小时；一般每天大概工作______小

**I3a3.5** 您一般每天几点开始工作？_______；工作结束时间是几点_______（按24小时制计算）

**I3a3.6** 您一般每个月休息 天； 上一个月实际休息了 天。

**I3a3.7** 在工作中，您的顾客/服务对象主要是以下的哪些机构/人员？

|  | 1.绝大部分 | 2.大部分 | 3.少部分 | 4.几乎没有 | 99998不适用 |
| --- | --- | --- | --- | --- | --- |
| a. 政府机构/人员 | 1 | 2. | 3 | 4. | 99998. |
| b. 事业单位/人员 | 1 | 2. | 3 | 4. | 99998. |
| c. 商业、企业机构/人员 | 1 | 2. | 3 | 4. | 99998. |
| d. 社会组织机构/人员 | 1 | 2. | 3 | 4. | 99998. |
| e. 散客 | 1 | 2. | 3 | 4. | 99998. |

**I3a3.8** 您和以上顾客/服务对象的业务/工作往来是否稳定?

|  | 1.非常稳定 | 2.比较稳定 | 3.不太稳定 | 4.很不稳定 | 99998不适用 |
| --- | --- | --- | --- | --- | --- |
| a. 政府机构/人员 | 1 | 2. | 3 | 4. | 99998. |
| b. 事业单位/人员 | 1 | 2. | 3 | 4. | 99998. |
| c. 商业、企业机构/人员 | 1 | 2. | 3 | 4. | 99998. |
| d. 社会组织机构/人员 | 1 | 2. | 3 | 4. | 99998. |
| e. 散客 | 1 | 2. | 3 | 4. | 99998. |

**I3a3.9** 在您看来，要做好这个工作，是否需要接受专门的训练或培训？

1.需要 2.不需要

**I3a3.10** 为掌握这份工作所需要的主要技能，您花了多少时间？（单选）

1.一天 2.几天 3.大约一周 4.不到一个月

5.一个月到三个月 6.超过三个月，不到一年 7.一年以上 8.三年以上

【CAPI】 if I3a3.1==99998，则跳至I3a4.6，否则继续询问

**I3a3.13** 您生意开始时注册资本／投入资金大约是：

1.5000元以下 2.5000元—1万 3.1—5万

4.5—10万 5.10-50万 6.50-100万

7.100万以上

**I3a3.14** 注册资本/投入资金是从哪些渠道筹集的? 依次写出最主要三项（见编码11）？

______\________\_______

1.个人储蓄 2.家人资助 3.其他亲戚 4.亲密朋友

5.一般朋友 6.生意伙伴 7.其他社会关系 8.银行商业性贷款

9.银行政策性贷款 10.风险投资 11.其他

**I3a3.15** 生意第一年竞争激烈吗？

1.非常激烈 2.比较激烈 3.不太激烈 4.根本不激烈 5.没有竞争

9.不好说

**I3a3.16** 您第一年所做的生意是否有来自下列渠道（多选 见编码14）？

1.党政机关 2.国有企业 3.国有事业 4.集体企业

5.个体经营 6.私营企业 7.外资/合资企业 8.股份制企业

9.个人/顾客 10.境外企业

**I3a3.17** 您生意开始时，有多少人主动给您提供生意项目？人数是_____

【CAPI】if I3a3.17=0或I3a3.17=99998，则跳至I3a3.19

**I3a3.18** 您与提供生意的人以前认识吗?（提供最重要的生意项目的那位）

1.认识 0.不认识**(跳至I3a3.19)**

**I3a3.18.1** 您与他/她是亲属吗？ 1.是 0.否

**I3a3.18.2** 您与他/她曾是同事吗？ 1.是 0.否

**I3a3.18.3** 那时您们的相熟程度是：

1.熟极了 2.很熟 3.较熟 4.不太熟 5.不熟

**I3a3.18.4** 您们之间亲密程度是:

1.亲密极了 2.很亲密 3.较亲密 4.不太亲密 5.不亲密

**I3a3.18.5**您们的信任程度是:

1.信任极了 2.很信任 3.较信任 4.不太信任 5.不信任

**I3a3.19** 到现在为止，给您介绍过生意的估计有______人？**【填答0跳至I3a3.22】**

**I3a3.20** 给您介绍过生意的人都包括下列哪些人？(多选 见编码15）

1.家人 2.亲属 3.亲密朋友 4.一般朋友

5.同乡 6.同学 7.战友 8.邻里

9.师生 10.师徒 11.同事 12.生意/项目伙伴 13.间接关系 14.其他（请说明） ________

15.没有人介绍 **(跳至I3a3.22)**

**I3a3.21** 他们之中有人在下列部门工作吗? (多选 见编码14)

1.党政机关 2.国有企业 3.国有事业 4.集体企业

5.个体经营 6.私营企业 7.外资/合资 8.股份制企业

9.工商联 10.企业家协会 11.行业协会 12.专业协会

**I3a3.22** 下列哪些说法符合您做生意时人际关系的作用?

**I3a3.22.1** 找了关系帮忙，但没起什么作用? 1.符合 2.不符合

**I3a3.22.2** 有的关系起了作用,但不是决定性的? 1.符合 2.不符合

**I3a3.22.3** 起关键作用的只有一个关系? 1.符合 2.不符合

**I3a2.23** 您个人现在在该生意上的资产总额有__________万元；

**I3a2.23.1** 您个人现在在该生意上负债________万元

**I3a2.23.2** 您2015年的全年利润总额大约是：_________万元

**I3a3.24** 2015年7月份以来您的生意竞争激烈吗？

1.非常激烈 2.比较激烈 3.不太激烈 4.根本不激烈 5.没有竞争

9.不好说

**I3a4.6** 您目前的工作需要专用的设备或工具吗？

1.不需要任何设备或工具；

2.需要,购买价格______________元

**I3a4.9** 自从您做这份工作以来，是否有跟政府有关部门（例如城管等）打过交道？

1.是 2.否 **(**跳至3B兼业部分**)**

**I3a4.10** 2015年7月以来，您在跟政府有关部门打交道的过程中,有没有发生过纠纷？

（例如：被拘留扣押，被勒令停业、店面被查封、与管理人员发生肢体冲突等）

1是 2否（跳至3B兼业部分）

**I3a4.10.3**这次纠纷最后是通过什么途径解决的？

1. 法律途径（如向法院起诉） 2.找工会

3.找对方上级领导或上级单位解决 4.找熟人调解 5.找媒体曝光 6.集体行动（如上访、罢工、散步等）

7.个体抗争（停工、上访等）

7.上网反映情况 8.没采取任何行动

9.其他（请注明）___________

**A5农业生产**

**I3a5.1** 对于您来说，请问农闲时间是在哪几个月？（多选题）

1.一月 2.二月 3.三月 4.四月

5.五月 6.六月 7.七月 8.八月

9.九月 10.十月 11.十一月 12.十二月

**I3a5.2** 2015年一年里，您用于农业生产的时间大概有多少天？______天

**I3a5.3.1** 2015年一年里，在农忙季节，您每天做几个小时的农活？_____小时

**I3a5.3.2** 2015年的一年里，在农闲季节，您每天做几个小时的农活？_____小时

**I3a5.4** 请问在2015年的农闲季节，您主要做什么？

1．务农 2 在家休闲 3 从事非农工作 4 其他（请注明）_______

【CAPI】 if I3a5.4=3,则继续询问I3a5.4.1至I3a5.4.3题。否则，跳至I3b.1题

**I3a5.4.1** 请问您的工作具体是什么？___________（若有多份工作，则问工作时间最长的那份）

**I3a5.4.2**您总共做了多少天这份工作？_______天

**I3a5.4.3** 您此份工作的工作地点在哪里？(可以多选)

1.本村居 2.本乡镇的其他村居 3.县/区内的其他乡镇 4.县城 5.本县/区之外的地方

【CAPI】回答完I3a5.4.3题，跳至 I3b.1题

**3B 兼业情况**

**I3b.1** 请问您目前除了从事* I3a.7*这份工作外，是否还有从事其他的有收入的工作？

1.是 2.否（跳至工作史部分）

**I3b.2**请问您目前从事的这份兼职工作的工作内容是？__________

**I3b.3**请问您目前从事的这份兼职工作的所属行业是？________

**I3b.4**请问您这份兼职工作是什么时候开始的？_______年______月

**I3b.5**请问您**平均每个月**能从这份兼职工作中获取的收入是_________元

**I3b.6**请问您**平均每周**要花在这份兼职工作中的时间是________小时

**I3b.7**请问您从事这份兼职工作最主要的原因是？

1.为提高收入

2.为了发挥自己具有的技能

3.为了投身于自己感兴趣的领域

4.为将来创业或将它作为主要职业做准备

**I3b.8**请问您这份兼职工作将来的打算是？

1.继续将它作为一份兼职 2.将它变为主要职业 3.放弃兼业

**(访问员注意：答完3B，跳至工作史部分)**

**3C、现在****无工作者**

【CAPI】 if work2015==2或I3a.7.0==2,则从I3c.1就开始询问;

if work==2,则从I3c.4才开始询问

**I3c.1** 您的上一份工作的职业是什么__________（失业前的这一份工作）

**I3c.2** 您的上一份工作是什么时候结束的？______年______月

**I3c.3** 您结束上一份工作的原因是（见编码16.1）？

1.家庭原因（包括怀孕、结婚、带孩子、家务劳动等）

2.返回学校 3.健康原因 4.退休

5.季节性、阶段性工作已经完成 6.合同到期 7.选择提早退休

8.企业/单位裁员 9.企业/单位倒闭 10.被辞退/开除

11.自己辞职 12.生意不好，放弃经营 13.年纪大了，身体状况不好

13.土地被征收 14.其他(请说明）_______

**I3c.4** 在无工作期间，生活费的主要来源是？（多选）

1.离退休费，请问您一个月退休费有多少元？__________元

2.下岗生活费, 请问您一个月下岗生活费有多少元？__________元

3.失业救济金, 请问您一个月失业救济金有多少元？__________元

4.社会救济 5.积蓄 6.亲友接济

7.临时性工作收入 8.其他家庭成员的收入 9.借债

10.租金收入 11.金融产品收入（股票、基金、债券等）

12.上学，由父母抚养 13.其他(请说明）_______

**I3c.5** 自从无工作后（有工作经历的人）//(无工作经历直接问)您有没有找过工作？

1.有**（跳至 3D无工作者的求职状况）** 2.没有

**I3c.6** 您没有找工作的主要原因是（见编码16）？**（最多选三项）**

1.没有合适的工作 2.找不到任何工作

3.缺乏学历/技能/经验 4.年龄太轻

5.年龄太老 6.其他种类的歧视

7.为了照顾小孩 8.为了照顾家中其他人

9.正在上学/培训 10.受自身健康状况的限制

11准备筹备创业 12.其他(请注明)_____

**I3c.7** 您现在想要一份工作（全职或兼职都可以）吗？

1.是 2.否 3.看情况

**I3c.8** 您是否打算在接下来的一年中找工作？

1.是 2.否 3.看情况

【CAPI】 if I3c.6==11，则询问

**I3c.9** 您的创业目标是什么？**________________**

【CAPI】if **I3c.5**==1,则继续回答**I3d.1；**if **I3c.5**==2,则跳至（四A）工作史部分

**3D、无工作者的求职情况**

**I3d.1** 无工作以来（如果无工作时间超过2年，问最近两年的情况）您都做了哪些与找工作相关的事（见编码17）？（多选）

1.直接与某雇主/自雇联系/面试 2.找职业中介机构

3.参加人才招聘会 4.找朋友或亲戚

5.找学校的就业指导中心 6.邮寄或网上发简历或申请

7.看相关广告 8.参加职业培训或课程

9.其他与找工作相关的活动 10.什么都没做

**I3d.2**无工作以来（如果无工作时间超过2年，问最近两年的情况）有人主动给您提供过求职方面的信息或帮助吗? 如果有，人数是_____（0=没有）

**I3d.3**无工作以来（如果无工作时间超过2年，问最近两年的情况）, 您大概主动找了多少人帮忙，包括打听就业信息，沟通情况等？____人

【CAPI】 if I3d.2和I3d.3都为0则跳至I3d.6

**I3d.4** 在您找工作的过程中，给您提供过帮助的人与您都有哪些关系？(多选)

1.家人 2.亲属 3.亲密朋友 4.一般朋友 5.同乡

6.同学 7.战友 8.邻里 9.师生 10.师徒

11.同事 12.间接关系 13.生意/项目伙伴 14.上下级领导关系

15.企业机构 16.其他（请说明） _________

**I3d.5** 他们帮助您做了什么? (多选 见编码18)

1.提供就业信息 2.告知招工单位/雇主的情况

3.提出具体建议,指导申请 4.帮助整理申请材料

5.亲自准备申请材料 6.帮助报名、递交申请

7.帮助推荐 8.帮助向有关方面打招呼

9.安排与有关人员见面 10.陪同造访有关人员

11.帮助解决求职中的具体问题 12. 直接提供工作

13.其他（请说明）___________________

**I3d.6** 上周，如果给您一个工作机会，您是否可以开始工作？ 1.是 2.否

**I3d.7** 无工作以来到上周末为止，您找了多长时间的工作？

**I3d.7.1** 如果是几周，那么有______周

**I3d.7.2** 如果是几个月，那么有________个月

**I3d.7.3** 如果是几年，那么有_________年

**I3d.8**无工作以来到上周末为止，您是否去过本县市以外的地方找过工作？

1是 2否（跳至I3d.9）

**I3d.8.1**去过哪些地方找工作？______省（直辖市）______市______县/区

**I3d.9** 您是不是在找一份全职工作（每周工作35小时以上）？

1.是 2.否 3.是不是全职工作不重要

**(四A) 工作史（新增部分回答，追访不答）**

**（访问员请注意：本部分对于所有的有过工作经历的人均需要访问）**

【CAPI】if work==1且work2015==2则从I4a.2.1开始；

if work==1且work2015==1则跳至I4a.2.4开始；

if work==2 则跳至第六部分；

**I4a.2.1** 您最近这份工作的具体职业是什么_______

格式“在（单位性质）（行业）做（具体职业名称）”，

如：在国有电子厂做办公室文员，在私营机械厂做流水线装配工，在街头摆摊卖水果等

**I4a.2.2**您在这份工作中最后那几个月的平均收入是多少？_______元

**I4a.2.3**您最近这份工作,所在的单位类型是什么（见编码8）？

（访问员注意：单位应该是一个独立的机构，有自己的财务和人事管理职权。如果受访者的工作机构分很多层级，无法区分哪一级是自己的单位时，可以提示，受访者工资关系所在的那一级，就可能是他/她的单位：由劳务派遣机构派出的保安，劳务工，家政服务员等，劳务派遣机构是其单位；个体经营者也要填答。如果被访者的工作没有单位，则填答10或11.）

【有单位】

1.党政机关、人民团体、军队 2.国有/集体事业单位 3.国营企业

4.集体企业 5.村居委会等自治组织 6.民营、私营企业

7.外资、合资企业 8 民办非企业、社团等社会组织

9.个体工商户（包括登记过的个体工商户或未登记的各类店主）

【无单位】

10.务农：农林牧副渔业生产（如种地、养殖鸡鸭水产等）

11.自由工作者（自由职业者，零散工，摊贩，无派遣单位的保姆，自营运司机，手工工匠等）

**I4a.2.4**您目前或最近这份工作是什么时候开始的：_____年

【CAPI】如果I3a.7.0=1自动生成结束年份为99998，跳至I4a.2.6；

如果I3a.7.0不等于1，则询问下一题

【Data】if I3a.7.0=1 生成I4a.2.2=99998

**I4a.2.5**您这份工作是哪一年结束的？________年

**I4a.2.10.w16**您离开这个工作单位是主动辞职吗？

1. 是 2.否

【CAPI】if I3a.9或I4a.2.3=1 2 3 4 5 8则询问单位归属 (只针对党政机关、国有企业和集体企事业单位)

**I4a.2.6**您目前或最近这份工作的单位归属是？

1中央部门所属 2省（直辖市）属 3市（地区）属 4县属

5街道乡镇所属 6村/居委会所属 99998不适用

【CAPI】if I3a.9或I4a.2.3= 10或11则跳过I4a.2.7-I4a.2.9

**I4a.2.7**您目前或最近这份工作的单位规模是？__________人

**I4a.2.8** 您在目前或最近这份工作单位中的主要职务是（见编码19）？

党务：11.单位党务负责人 12.单位中层党务干部 13.单位一般党务干部

行政：21.单位行政负责人 22.单位中层行政干部 23.单位一般行政干部

业务：31.单位业务负责人 32.单位中层业务干部 33.单位一般业务干部

其他：41.一般工作人员 99998.不适用

**I4a.2.9** 您在目前或最近这份工作的单位的行政级别是（见编码20）？

1.司局级(地区)以上 2.处级(县) 3.副处级

4.科级 5.副科级 6.股级

7.副股级 8.科员 9.村（居）委会负责人

99998不适用

**I4a.3.0** 刚刚谈的这份工作，是不是您的第一份工作？（即您工作以来是否只有过这一份工作）

1.是（跳至**流动意愿部分**） 2.否

**I4a.3.1** 您的第一份工作的具体职业是什么_______

格式“在（单位性质）（行业）做（具体职业名称）”，

如：在国有电子厂做办公室文员，在私营机械厂做流水线装配工，在街头摆摊卖水果等

**I4a.3.2.1** 您的第一份工作是哪一年开始的？_______年

**I4a.3.2.2**您的第一份工作是哪一年结束的？_______年

**I4a.3.8.w16**您离开这个工作单位是主动辞职吗？

1. 是 2.否

**I4a.3.2.3** 您在这份工作中最后那几个月的平均收入是多少？_______元

**I4a.3.3**您第一份工作* I4a.3.1*,所在的单位类型是什么（见编码8）？

（访问员注意：单位应该是一个独立的机构，有自己的财务和人事管理职权。如果受访者的工作机构分很多层级，无法区分哪一级是自己的单位时，可以提示，受访者工资关系所在的那一级，就可能是他/她的单位：由劳务派遣机构派出的保安，劳务工，家政服务员等，劳务派遣机构是其单位；个体经营者也要填答。如果被访者的工作没有单位，则填答10或11.）

【有单位】

1.党政机关、人民团体、军队 2.国有/集体事业单位 3.国营企业

4.集体企业 5.村居委会等自治组织 6.民营、私营企业

7.外资、合资企业 8 民办非企业、社团等社会组织

9.个体工商户（包括登记过的个体工商户或未登记的各类店主）

【无单位】

10.务农：农林牧副渔业生产（如种地、养殖鸡鸭水产等）

11.自由工作者（自由职业者，零散工，摊贩，无派遣单位的保姆，自营运司机，手工工匠等）

【CAPI】if I4a.3.3=10或11则跳过I4a.3.4-I4a.3.7

**I4a.3.4**您第一份工作* I4a.3.1*的职务是下列哪一种（见编码19）？

党务：11.单位党务负责人 12.单位中层党务干部 13.单位一般党务干部

行政：21.单位行政负责人 22.单位中层行政干部 23.单位一般行政干部

业务：31.单位业务负责人 32.单位中层业务干部 33.单位一般业务干部

其他：41.一般工作人员 99998.不适用

**I4a.3.5**您第一份工作* I4a.3.1*,您在目前或最近这份工作的单位的行政级别是（见编码20）？

1.司局级(地区)以上 2.处级(县) 3.副处级

4.科级 5.副科级 6.股级

7.副股级 8.科员 9.村（居）委会负责人

99998不适用

【CAPI】if I4a.3.3=1 2 3 4 5 8则询问单位归属 (只针对党政机关、国有企业和集体企事业单位)

**I4a.3.6**您第一份工作* I4a.3.1*,所在的单位归属是什么？(针对党政机关、国有企业和集体企事业单位)

1中央部门所属 2省（直辖市）属 3市（地区）属 4县属

5街道乡镇所属 6村/居委会所属 99998不适用

**I4a.3.7**您第一份工作* I4a.3.1*,单位规模是？_________人

【CAPI】 if I3a.7.0==1，则询问**I4a.4.0，**if I3a.7.0==2**跳至I4a.5.1**

**I4a.4.0**您的第一份工作就是您的前一份工作吗？（即您工作以来，是否只有过这两份工作经历）

1.是（跳至工作意愿部分） 2.否

**I4a.4.1**您目前工作的前一份工作的具体职业是什么_______

格式“在（单位性质）（行业）做（具体职业名称）”，

如：在国有电子厂做办公室文员，在私营机械厂做流水线装配工，在街头摆摊卖水果等

**I4a.4.2.1**您这份工作是哪一年开始的？_______年

**I4a.4.2.2**您这份工作是哪一年结束的？_______年

**I4a.4.8.w16**您离开这个工作单位是主动辞职吗？

1. 是 2.否

**I4a.4.2.3**您在这份工作中最后那几个月的平均收入是多少？_______元

**I4a.4.3**您前一份工作* I4a.4.1*,所在的单位类型是什么（见编码8）？

（访问员注意：单位应该是一个独立的机构，有自己的财务和人事管理职权。如果受访者的工作机构分很多层级，无法区分哪一级是自己的单位时，可以提示，受访者工资关系所在的那一级，就可能是他/她的单位：由劳务派遣机构派出的保安，劳务工，家政服务员等，劳务派遣机构是其单位；个体经营者也要填答。如果被访者的工作没有单位，则填答10或11.）

【有单位】

1.党政机关、人民团体、军队 2.国有/集体事业单位 3.国营企业

4.集体企业 5.村居委会等自治组织 6.民营、私营企业

7.外资、合资企业 8 民办非企业、社团等社会组织

9.个体工商户（包括登记过的个体工商户或未登记的各类店主）

【无单位】

10.务农：农林牧副渔业生产（如种地、养殖鸡鸭水产等）

11.自由工作者（自由职业者，零散工，摊贩，无派遣单位的保姆，自营运司机，手工工匠等）

【CAPI】if I4a.4.3=10或11，则跳过**I4a.4.4-I4a.4.7，跳至I4a.5.1**

**I4a.4.4**您前一份工作* I4a.4.1*的职务是下列哪一种（见编码19）？

党务：11.单位党务负责人 12.单位中层党务干部 13.单位一般党务干部

行政：21.单位行政负责人 22.单位中层行政干部 23.单位一般行政干部

业务：31.单位业务负责人 32.单位中层业务干部 33.单位一般业务干部

其他：41.一般工作人员 99998.不适用

**I4a.4.5**您在前一份* I4a.4.1*的工作单位里的行政级别是（见编码20）？

1.司局级(地区)以上 2.处级(县) 3.副处级

4.科级 5.副科级 6.股级

7.副股级 8.科员 9.村（居）委会负责人

99998不适用

【CAPI】if I4a.4.3=1 2 3 4 5 8则询问单位归属 (只针对党政机关、国有企业和集体企事业单位)

**I4a.4.6**您前一份工作* I4a.4.1*,所在的单位归属是什么？(针对党政机关、国有企业和集体企事业单位)

1中央部门所属 2省（直辖市）属 3市（地区）属 4县属

5街道乡镇所属 6村/居委会所属 99998不适用

**I4a.4.7**您前一份工作* I4a.4.1*,单位规模是？_________人

**I4a.5.1**您自工作以来，一共有过几次工作经历？________次

**流动意愿（村委会农村户籍者回答）**

【CAPI】 if Itype==1（村委会）,且户口为农业户籍，则询问I4b.1,否则跳至I10.1.w16

**I4b.1** 请问您有外出务工(跨县流动半年以上)经历？

1.是（跳至I4b.2.1） 2.否

**I4b.1.1** 请问您是否有到本县（区）其他乡镇（目前居住乡镇之外）连续务工时间超过6个月以上的经历?

1.是（跳至I4b.6） 2.否（跳至I4b.6）

**I4b.2.1** 请问您最近一次跨县外出是什么时候离开家乡的？________年

**I4b.2.2** 请问您这次是什么时候返回家乡的？________年_____月

**I4b.3** 请问您是否还准备外出务工吗？（外出指到其他县区工作）

1.是 2.否（跳至**I4b.4**）

I4b.3.1 您准备呆多少个月？_______月

**I4b.3.2** 请问您这次回来的原因是什么（见编码21）？

1.节假日返乡 2.农忙回来帮忙 3.为了参加婚丧嫁娶等红白喜事

4.看望家人 5.暂时不想工作，回家休息 6.结束上一份工作，想找更好的工作

7.相亲 8.回来生小孩 9.生病，受伤等身体因素限制

10.其他（请注明）______

**I4b.4** 您打算长期呆在家乡的原因是（见编码22）？

1.为了照顾家人 2.回家乡也能获得较好的收入

3.在外找不到好工作 4.习惯了本地生活，不习惯外地生活

5.生病，受伤等身体因素限制 6.其他（请注明）______

【CAPI】 if I4b.4==3 ，则询问I4b.4.1，否则跳至I4b.4.2

**I4b.4.1** 请问在外找不到好工作的原因是？

1.年纪太大 2.文化、技能等自身能力低

3.生病，受伤等身体因素限制 4.相关工作领域不需要我的技能

5.其他（请注明）________

**I4b.4.2**请问您这次回来，是一个人回来的，还是带家人一起回来的？

1.一个人回来 2.和家人一起回来 3.没有其他家人外出

**I4b.4.3** 请问您在村里是否还有土地？ 1.是 2.否

**I4b.4.4** 您在村里是否购房或者建房？ 1.是 2.否

**I4b.4.5** 请问如果村里有分红或其他的利益分配，您是否能够分得到？ 1.能 2.不能

**I4b.4.6** 您在家乡是否有投资经营活动？ 1.是 2.否

**I4b.4.7** 请问您接下来的打算是？

1.在村里务农 2.在村里从事其他的非农工作 3.到镇上或县城工作

**I4b.5** 请问您一共在多少个省份（包括本省）工作过？（出省旅游、探亲和出差都不算）____个

【CAPI】if I4b.1==2，则询问I4b.6，否则跳至I4b.7

**I4b.6** 请问您是否打算外出务工？ 1.是 2.否（跳至I4b.7）

**I4b.6.1** 请问您打算将来外出务工吗？

1.年内准备出去 2.打算1-2年内出去 3.打算3-5年内出去

4.五年之后再打算 5.不打算出去了 6.不适用（目前在外务工）

**I4b.7** 未来5年，你是否计划到城镇定居？

1.是 2.否 3.已经在城镇定居

**I4b.8** 未来5年，你是否计划在城镇建房或者购房？

1.是 2.否（跳至I10.1.w16） 3.已经在城镇建房或者购房

**I4b.9** 您在城镇建房或购房的主要目的是？

1.在城镇定居生活 2.投资

3.为了子女定居城市准备 3.为了子女教育

4.其他（请注明）_________

**十w16、无业史**

【CAPI】if work=2,跳至 五:创业过程;

If work=1且I3a.7.0=1，则问I10.1a.w16

I10.1a.w16自1990年以来（接受全日制教育期间不算），您一共有过几次没有工作的经历？______次（毕业后没工作后也算）。

（这里没有工作的经历只有超过3个月才计算。工作指的是从事有收入的活动，务农、兼职、帮忙家庭生意也算在内，不包括义工、学生兼职、志愿者、家务劳动这些活动）

【CAPI】If work=1且I3a.7.0=2，则问I10.1b.w16

I10.1b.w16**除目前这次无工作外，**自1990年以来（接受全日制教育期间不算），您一共有过几次没有工作的经历？______次（毕业后没工作后也算）。

（这里没有工作的经历只有超过3个月才计算。工作指的是从事有收入的活动，务农、兼职、帮忙家庭生意也算在内，不包括义工、学生兼职、志愿者、家务劳动这些活动）

【CAPI】if I10.1a.w16=0或I10.1b.w16=0,跳至 五:创业过程

I10.2.w16请您告诉我们每次不工作的情况：

| 1.次数 | 2.开始时间  （年月） | 3.结束时间  （年月） | 4.无工作前的工作的单位类型（编码8） | 5.无工作前的工作的行业  （编码37） | 6.结束上一份工作的原因 | 7.是否获得国家或单位的生活保障金  1是 2否 | 8.之后参加工作的单位类型  （编码8） | 9.之后参加工作的行业  （编码37） |
| --- | --- | --- | --- | --- | --- | --- | --- | --- |
| 1 |  |  |  |  |  |  |  |  |
| 2 |  |  |  |  |  |  |  |  |
| …… |  |  |  |  |  |  |  |  |

您结束上一份工作的原因是（见编码16.1）？

1.家庭原因（包括怀孕、结婚、带孩子、家务劳动等）

2.返回学校 3.健康原因 4.退休

5.季节性、阶段性工作已经完成 6.合同到期 7.选择提早退休

8.企业/单位裁员 9.企业/单位倒闭 10.被辞退/开除

11.自己辞职 12.生意不好，放弃经营13.土地被征收 14.其他(请说明）_______

**（五）****创业过程**

【CAPI】if I3a.16=1或（ I3a.16=3且 I3a3.1=99998） 或 I3a.16=4则回答Id.1

（说明：现在不是雇主或个体店主的，询问曾经的创业尝试）

**Id.1**您曾经尝试创业吗？ 1.是（继续） 2.否（跳至**I6.1** ）

【CAPI】if I3a.16=2或（ I3a.16=3且 I3a3.1！=99998 ）

（说明：现在是雇主或个体店主的，询问现在这次创业之外的其他创业尝试）

**Id.1.1**您除了这次现在创业外，还有过其他创业尝试吗？ 1.是（继续） 2.否（结束）

**Id.2**您一共的创业次数是____次（包括目前的创业）；其中赚到钱____次，不赚不赔____次，赔钱____次

**Id.3**您是什么时候第一次创业/做生意的？_______年

**Id.4**您第一次创业所属行业

**Id.5**您创业是因为找到好的创业机会还是因为您没有更好的工作选择？

【读出】

1.抓住好的创业机会 2.没有更好的工作选择

【不读出】

3.以上两个都是 4.当时有好的工作岗位，但创业机会更加好

【CAPI】 if Id.5==1或Id.5==3或Id.5==4, 则询问Id.6

**Id.6**您的创业机会来自于下列哪种渠道？（多选）

1.原来的工作经历 2.自己的发现

3.朋友的介绍 4.对海外企业的模仿和借鉴（？）

5.模仿身边的其他企业 6其他，请注明.

**Id.12.w16**您认为以下因素对您创业的重要程度如何？请在最能代表您真实意见的数字上打勾。

|  | 非常不重要 | 不重要 | 一般 | 重要 | 非常重要 |
| --- | --- | --- | --- | --- | --- |
| 有技术（如：有相关研究成果或专利） | 1 | 2 | 3 | 4 | 5 |
| 有经营经验 | 1 | 2 | 3 | 4 | 5 |
| 有人脉关系支持 | 1 | 2 | 3 | 4 | 5 |
| 有经济基础 | 1 | 2 | 3 | 4 | 5 |
| 有政府支持 | 1 | 2 | 3 | 4 | 5 |

【CAPI】 if Id.5==2或Id.5==3, 则询问Id.7

**Id.7** 您第一次创业时,没有更好的工作选择的具体原因是（见编码13）?

1.文化、技能等自身能力低 2.相关工作领域不需要我的技能

3.失去土地 4.生病，受伤等身体因素限制

5.年纪太大 6.其他（请注明）________

**Id.8** 您第一次创业是否结束？

1..否（跳至**I6.1**） 2..是

**Id.9** 您是什么时候结束这次创业的？______年

**Id.10** 请问您第一次创业终止是在哪一个阶段？

1. .刚刚有了创业的初步规划（概念期）

2..开始筹备产品或服务（创业早期）

3..已经有了销售或生产收入，但尚未真正盈利（成长期）

4..已有可观的销售收入，并有较多利润（扩展期）

5..已有稳定利润并有进一步发展规划（成熟期）

**Id.11** 您第一次创业的项目或生意终止的最重要原因是？

1.有一个出售这个项目或生意的好机会 2.这个项目或生意不赚钱

3.有其他工作或创业机会 4.缺乏资金投入

5.在开始这个项目前就计划要退出 6.创业太艰辛、压力太大

7.合伙人矛盾或退出 8.发生意外事故（如天灾或合伙人重病）

9.其他（请注明）______

**(六）社会参与与支持**

**I6.1** 在本地，您有多少[关系](http://www.5xx.cn/data/25461/)密切，可以得到他们支持和帮助的朋友/熟人？________个

**I6.1.1** 在本地这些关系密切的人中，您可以向他/她诉说心事的有几个？________个

**I6.1.2** 在本地这些关系密切的人中，您可以同他/她讨论重要问题的有几个？________个

**I6.1.3** 在本地这些关系密切的人中，您可以向他/她借钱（5000元为标准）的有几个？___个

**I6.3** 您和本社区（村）的邻里, 街坊及其他居民互相之间的熟悉程度是怎样的？

1.非常不熟悉 2.不太熟悉 3.一般 4.比较熟悉 5.非常熟悉

**I6.4** 您对本社区（村）的邻里, 街坊及其他居民信任吗？

1.非常不信任 2.不太信任 3.一般 4.比较信任 5.非常信任

**I6.5** 您与本社区（村）的邻里, 街坊及其他居民互相之间有互助吗？

1.非常少 2.比较少 3.一般 4.比较多 5.非常多

I6.5.1 你觉得你所在的社区安全吗？

1□ 很安全 2□ 较安全 3□ 不太安全 4□ 很不安全

**I6.6** 在本村/居委会上次的选举中，您是：

1.自己去投票 2.家人代投票 3.没去投票 99998.不适用

**I6.7** 社团、社会组织参与状况(请在相应的选项打“√”) （仅城市社区）

| I6.7.1序号 | I6.7.2您是否是其成员？  1.是  2.否**（跳至下一项）** | I6.7.3过去一年，您参加该组织活动的频率怎样？  1.每天 2.一周数次 3.一月数次 4.一年数次或更少 5.从不 |
| --- | --- | --- |
| a.居委会 | -- |  |
| b社工机构 |  |  |
| c.业主委员会 |  |  |
| d.休闲/娱乐/体育俱乐部/沙龙组织 |  |  |
| e.学习/培训机构 |  |  |
| f.同乡会 |  |  |
| g宗亲组织 |  |  |
| h.公益/社会组织/志愿者团体 |  |  |
| i.宗教组织 |  |  |

**I6.8** 过去三个月您在外就餐的情况：(单选)

|  | 从不 | 很少 | .有时 | 较多 | 经常 | 不适用 |
| --- | --- | --- | --- | --- | --- | --- |
| I6.8.1 工作日在外晚餐吗？ | 1 | 2. | 3 | 4. | 5. | 99998. |
| I6.8.2 休息日在外晚餐吗？ | 1 | 2. | 3 | 4. | 5. | 99998. |
| I6.8.3 您请人在外就餐过吗？ | 1 | 2. | 3 | 4. | 5. | 99998. |
| I6.8.4 您被请在外就餐过吗？ | 1 | 2. | 3 | 4. | 5. | 99998. |
| I6.8.5 您陪朋友在外就餐过吗？ | 1 | 2. | 3 | 4. | 5. | 99998. |

**I6.9** 过去十二个月，你在本地有过下列经历吗？

|  | 1.有 | 2.没有 |
| --- | --- | --- |
| **I6.9.1** 被别人殴打 |  |  |
| **I6.9.2** 被别人诈骗财物 |  |  |
| **I6.9**.3 被别人偷窃 |  |  |
| **I6.9.4** 被别人恐吓勒索 |  |  |
| **I6.9.5** 被别人抢劫 |  |  |

**I6.10**未来五年里，您遭遇以下事件的可能性有多大？ (每行单选)

|  | 可能性非常大 | 可能性比较大 | 可能性比较小 | 可能性非常小 | 不知道/说不清 |
| --- | --- | --- | --- | --- | --- |
| 1.失业 | 1 | 2 | 3 | 4 | 9 |
| 2.犯罪侵害 | 1 | 2 | 3 | 4 | 9 |
| 6.遭遇恐怖袭击 | 1 | 2 | 3 | 4 | 9 |
| 7.吃到假药或伪劣食品 | 1 | 2 | 3 | 4 | 9 |
| 8.被传染某种传染病 | 1 | 2 | 3 | 4 | 9 |
| 9.遭遇环境污染问题 | 1 | 2 | 3 | 4 | 9 |

【CAPI】 if I1.14=1回答I6.22.w16-I6.22.2.w16，否则跳至I6.11

**I6.19.w16** 你是否接受过由政府提供的或者有政府补贴的职业技能培训？

1.是 2.否（跳至**I6.11** ）

**I6.19.1.w16**你接受过几次这样的培训？_______次

**I6.19.2.w16**你每一次接受培训的具体情况是怎样的？

| 次数 | **I6.19.2a.w16**.培训类型是？  1.就业技能培训  2.岗位技能提升培训  3.创业培训  99.其他 | **I6.19.2b.w16**..培训提供方或政府补贴提供方是哪一类？   1. 户籍所在地政府 2. 非户籍所在地政府   99999.不清楚 | **I6.19.2c.w16**..该培训是否对你有帮助？  1.是 2.否 |
| --- | --- | --- | --- |
| 1 |  |  |  |
| 2 |  |  |  |
| …… |  |  |  |

【CAPI】 if I1.3.2>=3，判断为流动人口，询问I6.11- I6.18，否则跳至I7.1

**I6.11** 您的工作单位是以本地人为主吗？

1.是 2.否**（跳至I6.12）** 99998.不适用 **（跳至I6.12）**

**I6.11.1** 您和工作单位的本地人交往频率是：

1.从不 2.偶尔 3.有时 4.经常

**I6.12**您居住的社区是以本地人为主吗？

1.是 2.否**（跳至**I6.19.w16 **）** 99999.不清楚**（跳至**I6.19.w16 **）**

**I6.12.1** 您和您居住社区的本地人交往频率是：

1.从不 2.偶尔 3.有时 4.经常

**I6.20.w16** 除上班时间外，您在本地平时与谁来往最多？ （ ）

1一起出来工作的亲戚 2一起出来工作的同乡 3 本地户籍亲戚

4 其他一起工作的朋友 5本地户籍同事 6政府管理服务人员

7本地同学/朋友（不包括同事） 8跟人来往不多 9其他人（请注明） _______

**I6.21.w16** 在本地遇到困难时，您一般向谁求助？ （ ）

1一起出来工作的亲戚 2一起出来工作的同乡 3本地户籍亲戚

4其他一起工作的朋友 5本地户籍同事 6行政执法部门人员

7本地同学/朋友（不包括同事） 8村/居委会、物业人员、房东 9很少找人

10 其他人（请注明） _______________

**I6.13** 您的本地方言水平掌握程度？

1.完全掌握 2掌握大部分 3.掌握部分 4.掌握一点点 5.根本不会

**I6.14** 您未来可能会在本地定居吗？

1.非常可能 2.比较可能 3.不确定 4.比较不可能 5.非常不可能

**I6.14.1** 如果您不打算在本地长期定居，最主要的原因是？

1.生活成本高 2.房价太高 3.小孩上学难 4.家人需要照顾

5.工作机会少 6.工作收入低 7.其他（请注明_______）

**I6.14.2** 如果您不打算在本地长期定居，那您将来最愿意在哪里定居（见编码26）：

1.老家农村 2.老家的镇上 3.老家的县城或地级市 4.老家的省会城市

5.其他中小城市 6.其他大城市 7.不清楚 8.其他（请注明___）

**I6.14.3**.您是否愿意放弃原来的户口，换取目前工作地的户口

1.不愿意 2.愿意 3.无所谓

【CAPI】农村户籍人口询问I6.15-I6.22.w16

**I6.15**您在老家是否还有土地？ 1.有 2.没有【跳至**I6.17**】

**I6.16**您是否愿意把户口迁入城市，？

1.不愿意（跳至I6.17） 2.愿意 4.说不清（跳至I6.17）

**I6.16.1** 如果可以把户口迁入城市，您是否愿意放弃老家的土地、集体分红等其他收益

1.不愿意 2.愿意 4.说不清

**I6.16.2** 如果可以把户口迁入城市，您最想要迁入的地点是？

1.现在打工的城市（跳至I6.17） 2.其他城市

**I6.16.3** 如果您不想把户口迁入现在打工的城市，那您最想要迁入的地点是？

1.家乡的乡镇 2家乡的县城. 3.家乡附近的中等城市

4.家乡的省会 5.外省的城市

**I6.17** 您在老家是否还有住房？ 1.有 2.没有

**I6.18** 如果老家的村集体进行分红或其他利益分配，您是否还能够分到？ 1.能 2.不能

**I6.22.w16** 您的父母是否也曾经像您一样离开家乡，进城务工或经商？ 1.是 2.否

**(七)劳动者状态**

**I7.1** 您信的是什么教？

1.天主教 2.基督教 3.佛教 4.藏传佛教 5.道教

6.伊斯兰教 7.东正教 8.其他宗教 9.无宗教信仰

**I7.2** 自2015年7月以来，您大概参加过多少从宗教仪式或活动？________次

【CAPI】 if work==1,则询问I7.3，否则跳至I7.14.w16

**I7.3** 请您对您目前/最后一份的工作状况进行评价：

| 项目 | 非常满意 | 比较满意 | 一般 | 不太满意 | 非常不满意 | 不适用 |
| --- | --- | --- | --- | --- | --- | --- |
| I7.3.1收入 | 1 | 2. | 3 | 4. | 5. | 99998. |
| I7.3.2工作安全性 | 1 | 2. | 3 | 4. | 5. | 99998. |
| I7.3.3工作环境 | 1 | 2. | 3 | 4. | 5. | 99998. |
| I7.3.4工作时间 | 1 | 2. | 3 | 4. | 5. | 99998. |
| I7.3.5晋升机会 | 1 | 2. | 3 | 4. | 5. | 99998. |
| I7.3.6工作有趣 | 1 | 2. | 3 | 4. | 5. | 99998. |
| I7.3.7工作合作者 | 1 | 2. | 3 | 4. | 5. | 99998. |
| I7.3.8能力和技能使用 | 1 | 2. | 3 | 4. | 5. | 99998. |
| I7.3.9他人给予工作的尊重 | 1 | 2. | 3 | 4. | 5. | 99998. |
| I7.3.10在工作中表达意见的机会 | 1 | 2. | 3 | 4. | 5. | 99998. |
| I7.3.11对工作的整体满意度 | 1 | 2. | 3 | 4. | 5. | 99998. |

**I7.4 目前工作**对你的意义或价值是什么？（如果目前为无工作，则询问的是最近这份工作）

|  | 1.非常符合 | 2.比较符合 | 3.无所谓 | 4.比较不符合 | 5.非常符合 |
| --- | --- | --- | --- | --- | --- |
| **I7.4.**1谋生 |  |  |  |  |  |
| **I7.4.**2让自己心安 |  |  |  |  |  |
| **I7.4.**3认识更多的人 |  |  |  |  |  |
| **I7.4.**4获得尊重 |  |  |  |  |  |
| **I7.4.5**兴趣 |  |  |  |  |  |
| **I7.4.**6充分发挥自己能力 |  |  |  |  |  |

【CAPI】 if utype==1（农村社区）,则询问I7.14.w16，if utype==2跳至I7.20.w16

I7.14.w16 你对下列说法是否赞成？

| 项目 | 非常赞同 | 比较赞同 | 一般 | 不太赞同 | 非常不赞同 |
| --- | --- | --- | --- | --- | --- |
| I7.14.1.w16 家里生男孩比生女孩好 | 1 | 2. | 3 | 4. | 5. |
| I7.14.2.w16 多子（女）多福 | 1 | 2. | 3 | 4. | 5. |
| I7.14.3.w16 上大学越来越没有用了 | 1 | 2. | 3 | 4. | 5. |
| I7.14.4.w16 相对于其他村民，我会积极采用新推广的农业技术 | 1 | 2. | 3 | 4. | 5. |
| I7.14.5.w16 即使不缺钱，也应该省着点花钱 | 1 | 2. | 3 | 4. | 5. |
| I7.14.6.w16 我不会向银行贷款去城镇买房 | 1 | 2. | 3 | 4. | 5. |
| I7.14.7.w16 外出打工比在家务农好 | 1 | 2. | 3 | 4. | 5. |
| I7.14.8.w16 农业生产对你家来说越来越不重要 | 1 | 2. | 3 | 4. | 5. |
| I7.14.9.w16 我越来越不适应农村生活 | 1 | 2. | 3 | 4. | 5. |

I7.15.w16 村里亲戚朋友办喜事，你会去送礼吗？

1.全部都去 2.大部分会去 3.少部分会去 4.极少会去

I7.16.w16 村里其他人（不是亲戚朋友）办喜事，你会去送礼吗？

1.全部都去 2.大部分会去 3.少部分会去 4.极少会去

I7.17.w16 假如村里组织修路，需要劳力/资金，你是否愿意参加/捐款？

1非常愿意 2比较愿意 3一般 4不愿意 5非常不愿意

I7.18.w16 假如村里组织投票选村干部，你是否愿意参加？

1非常愿意 2比较愿意 3一般 4不愿意 5非常不愿意

I7.19.w16 你是否赞同你的孩子去参军？

1、非常赞同 2、赞同 3、无所谓 4、不赞同 5、非常不赞同

I7.20.w16 现在中国的经济结构是多种所有制并存，你更接受下面哪种状况？

1.完全私有制 2大部分私有制 3各一半 4.大部分公有制 5.完全公有制

I7.21.w16 如果让你找（换）一份工作，你想去下列哪种部门工作？（单选）

1.党政机关、人民团体、军队 2.国有/集体事业单位 3.国营企业（含国有控股）

4.集体企业 5.村居委会等自治组织 6.民营、私营企业（含民营控股） 7.外资企业（含外资控股） 8.社会服务机构（民办非企业）、社团等社会组织

9.自由职业

I7.21.1.w16 您想去该类部门工作的原因是？（多选）

1.对这类单位很认可 2.在这类单位工作社会地位高 3.收入或福利高

4.工作稳定 5.工作时间合理 6.工作压力小

7.晋升机会多 8.工作有趣 9.可以发挥充分个人的能力和技能

11.在工作中表达意见的机会好 12.工作平台广 13.其他

【CAPI】if work2015==1,则询问I7.5，否则跳至I7.6

**I7.5**请您根据自己的感受和体会，判断以下状态在您身上发生的频率

|  | **1.每天** | **2.一周数次** | **3.一月数次** | **一年数**  **次或更少** | **5.从不** |
| --- | --- | --- | --- | --- | --- |
| **I7.5.1** 工作让我  感觉身心俱疲 |  |  |  |  |  |
| **I7.5.2** 整天工作对我  来说确实压力很大 |  |  |  |  |  |
| **I7.5.3** 我觉得自己完成了很多有价值的工作 |  |  |  |  |  |
| **I7.5.4** 我对这份  工作越来越不感兴趣 |  |  |  |  |  |

**I7.6** 幸福感

**I7.6.1** 总的来说，您认为您的生活过得是否幸福？

非常不幸福1------2------3------4------5非常幸福

I7.6.2 总体来说，您对您的生活状况感到满意么？

非常不满意1------2------3------4------5非常满意

I7.6.3 总体来说，您对您的家庭经济状况感到满意么？

非常不满意1------2------3------4------5非常满意

**I7.8** 请考虑下面的一些描述，您是否同意？

| 描述 | 非常不同意 | 不同意 | 同意 | 非常同意 |
| --- | --- | --- | --- | --- |
| **I7.8.1**就算身体有点不舒服，或者有其他理由可以休息，我也会努力完成每日应该做的事。（包括所有工作、学业及日常生活事务等）. | 1 | 2. | 3 | 4. |
| **I7.8.2**就算是我不喜欢的事，我也会尽全力去做。（包括所有工作、学业及日常生活事务等） | 1 | 2. | 3 | 4. |
| **I7.8.3**就算一件事需要花好长时间才能有结果，我仍然会不断地尽力去做。 | 1 | 2. | 3 | 4. |

**I7.9** 您认为您目前的生活水平和您在工作上的努力比起来是否公平？

1.完全不公平 2.比较不公平 3.说不上公平但也不能说不公平

4.比较公平 5.完全公平

**I7.9.1** 有一些人觉得可以完全选择自己的生活，而有一些人则觉得对于发生在自己身上的事无能为力。您觉得您选择自己生活的自由程度如何？请在1到10之间给自己打一个分，1表示完全没有选择权，10表示有很大的选择权。

| 完全没选择权 | |  | 有很大的选择权 | | |  |
| --- | --- | --- | --- | --- | --- | --- |
|  |  | 1 2 3 4 5 6 7 8 9 10 | |  |  | |

**I7.10** 在我们的社会里，有些人居于顶层，有些人则在底层。下面这种卡片上有一个从上往下的梯子，最高的“10”分代表最顶层，最低的“1”分代表最底层。


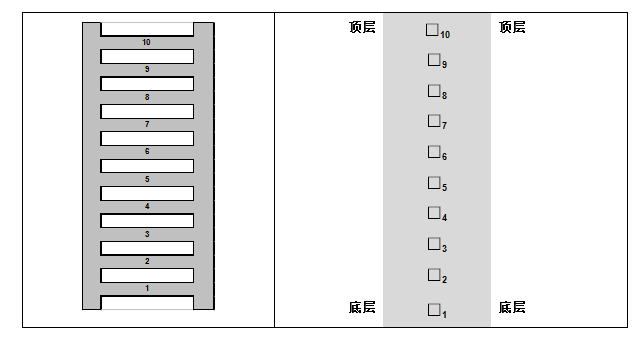


**（访问员注意：“10”分代表最顶层，“1”分代表最底层）**

**I7.10.1** 您认为您自己目前在哪个等级上？

记录：[____|____]分

**I7.10.2** 您认为您5年前在哪个等级上？

记录：[____|____]分

**I7.10.3** 您认为您5年后将会在哪个等级上？

记录：[____|____]分

**I7.10.4** 您认为在您14岁时，您的家庭处在哪个等级上？

记录：[____|____]分

您认为自己当前的生活水平与下列人相比，是好些还是差些？

|  | 低很多 | 低一些 | 差不多 | 高一些 | 高很多 |
| --- | --- | --- | --- | --- | --- |
| **I7.10.12**亲戚 | 1 | 2. | 3 | 4. | 5. |
| **I7.10.13**老同学 （教育水平和你一样） | 1 | 2. | 3 | 4. | 5. |
| **I7.10.14**邻居 | 1 | 2. | 3 | 4. | 5. |
| **I7.10.15**所在市辖区（或县）其他居民 | 1 | 2. | 3 | 4. | 5. |

**I7.11** 总的来说，您是否同意大多数人是可以信任的这种看法？

1.非常不同意 2.不同意 3.同意 4.非常同意

**I7.11.**1.w16对于下面几类人，您的信任程度怎样？

|  | 完全不可信 | 比较不可信 | 居于可信与不可信之间 | 比较可信 | 完全可信 |
| --- | --- | --- | --- | --- | --- |
| **I7.11.**2.w16 家人 |  |  |  |  |  |
| **I7.11.**3.w16 亲戚朋友 |  |  |  |  |  |
| **I7.11.**4.w16 邻居 |  |  |  |  |  |
| **I7.11.**5.w16 同学 |  |  |  |  |  |
| **I7.11.**6.w16 同乡 |  |  |  |  |  |
| **I7.11.**7.w16 陌生人 |  |  |  |  |  |
| **I7.11.**8.w16 一起工作或做事的人 |  |  |  |  |  |
| **I7.11.**9.w16 买东西接触到的生意人 |  |  |  |  |  |
| **I7.11.**10.w16 那些有宗教信仰的人 |  |  |  |  |  |

**I7.12** 您未来2年内有何打算（最多选两项 见编码27）

1.找一份新工作 2.继续目前工作/上学 3.离职脱产参加培训

4.离职生育子女 5.参加在职培训 6.离职/半职回家照顾家人；

7.退休 8.没考虑过/维持原状 9.暂时离职一段时间再继续工作

10.创业 11 .其他(请说明)______

【CAPI】if I7.12==1，则询问I7.13，否则跳过此题

**I7.13** 您打算做一份什么样的新工作？___________（填文字，大概描述下想做什么，职位，岗位）

**（八）生殖生育**

**（注：I8.1、I8.2只问52岁以下妇女）**

I8.1**月经史：**

I8.1.1 您多少岁时月经初潮？ 岁

I8.1.2是否绝经（自最末一次月经计起停经超过1年为绝经）？

1□. 是 最后一次月经时为 岁（请跳至I8.2） 2□. 否

I8.1.3近一年来月经是否正常？ 1□.是（请跳至I8.2） 2□. 否（请填I8.1.4）

（月经正常即月经周期有规律、经血量正常、经血颜色暗红、无血块）

I8.1.4是否因为痛经严重而就医？ 1□.是 2□. 否

I8.2**怀孕史**

I8.2.1您是否有不孕史（夫妇同居两年以上，配偶生殖功能正常，未采取避孕措施而不受孕）

1□.有 2□. 无

I8.2.2您曾经怀孕（包括流产、死产等情况）多少次？ 次（怀孕中算1一次，怀孕中妇女可以直接跳至I8.3；若是0跳至I8.5）

I8.2.3您怀孕的结果是否出现过异常(流产、早产、死产、难产)？

1□.是（请继续填写I8.2.4） 2□. 否（跳至I8.2.5）

I8.2.4 如有异常情况，属于下面哪种异常？

1.流产 2.早产 3.死产 4.死胎 5.难产

I8.2.5. 您生过几个孩子？ 个（若是0跳至I8.5）

您的第一个子女出生时，您的年龄？ 岁

您的第二个子女出生时，您的年龄？ 岁

I8.2.6您生过孩子中是否有先天性疾病或缺陷？

1.是 （如有，请继续填，有几个填几个） 2.否（跳至**I8.4.1.w16**）

缺陷儿是您第 次怀孕？ 疾病或缺陷的名称

缺陷儿是您第 次怀孕？ 疾病或缺陷的名称

**I8.4.1.w16** 您现在有______个男孩**I8.4.2.w16** _______个女孩？

【CAPI】if 年龄小于52岁，则询问I8.5 - I8.7，否则跳过此题

**I8.5** 您还打算要几个孩子？

1.不想再要了 2.再要一个 3.再要两个 4.再要三个及以上 5.还没想好

**I8.6**如果不考虑计划生育政策和经济、健康等条件，您认为**一个家庭通常**几个孩子最理想？

记录：____个； 其中，男____个；女____个

**I8.7**您认为两个孩子之间的出生间隔多少年合适？

1. 两年以内 2.3-5年 3.5-7年 4.7年以上 5.还没想好

**（九）健康状况**

**I9.1** 身高________厘米

**I9.2** 体重________斤**（注意：单位是斤(500克)，不是公斤(1000克)）**

**I9.3.2** 腰围 1.被访者拒绝自测或他测 2.已测 为_______厘米

**I9.3.3** 臀围 1.被访者拒绝自测或他测 2.已测 为_______厘米

**I9.3.5** 请访问员在完成肺功能监测之后记录肺功能监测数值：

**I9.3.5.1** 第1秒用力呼气容积：______

**I9.3.5.2** 请6秒用力呼气容积：______

I9.4.1 您认为自己现在的健康状况如何？

1.非常健康 2.健康 3.一般 4.比较不健康 5.非常不健康

I9.4.2 在过去一个月内，是否有过身体疼痛现象？

1.没有 2.很少 3.有时 4.经常 5.总是

I9.4.3 在过去一个月内，是否由于身体疼痛问题影响到您的工作或其他日常活动？

1.没有 2.很少 3.有时 4.经常 5.总是

I9.4.5.w16 在过去一个月中，是否出现过情绪问题（如感到沮丧或焦虑）？

1.没有 2.很少 3.有时 4.经常 5.总是

I9.4.4 在过去一个月内，是否由于情绪问题(如感到沮丧或焦虑)影响到您的工作或其他日常活动？

1.没有 2.很少 3.有时 4.经常 5.总是

I9.28.w16 过去的一周里，选择您出现的以下情况的频率：

|  | 项目 | 没有/基本没有  (少于1天) | 少有  (1-2天) | 常有  (3-4天) | 几乎一直有  (5-7天) |
| --- | --- | --- | --- | --- | --- |
|  | I9.28.1.w16 因一些小事而烦恼 |  |  |  |  |
|  | I9.28.2.w16 不想吃东西，胃口不好 |  |  |  |  |
|  | I9.28.3.w16 即使有家人和朋友帮助仍然无法摆脱心中苦闷 |  |  |  |  |
|  | I9.28.4.w16 觉得不如多数人好 |  |  |  |  |
|  | I9.28.5.w16 做事时无法集中注意力 |  |  |  |  |
|  | I9.28.6.w16 感到情绪低落 |  |  |  |  |
|  | I9.28.7.w16 感到做任何事都很费力 |  |  |  |  |
|  | I9.28.8.w16 感到前途没有希望 |  |  |  |  |
|  | I9.28.9.w16 觉得自己的生活是失败的 |  |  |  |  |
|  | I9.28.10.w16 感到害怕 |  |  |  |  |
|  | I9.28.11.w16 睡眠不好 |  |  |  |  |
|  | I9.28.12.w16 感到不高兴 |  |  |  |  |
|  | I9.28.13.w16 比平时说话要少 |  |  |  |  |
|  | I9.28.14.w16 感到孤单 |  |  |  |  |
|  | I9.28.15.w16 觉得人们对自己不太友好 |  |  |  |  |
|  | I9.28.16.w16 觉得生活没有意思 |  |  |  |  |
|  | I9.28.17.w16 曾哭泣 |  |  |  |  |
|  | I9.28.18.w16 感到忧愁 |  |  |  |  |
|  | I9.28.19.w16 感到人们不喜欢自己 |  |  |  |  |
|  | I9.28.20.w16 觉得生活无法继续下去 |  |  |  |  |

**I9.8**您是否有吸烟历史（即每天≧1支，连续吸1年及以上）

1□. 是 2□. 否（跳至**I9.9**）

I9.8.1您吸烟_________年

I9.8.2您吸烟的时候，平均每天吸多少支？ ____支/每天（如果被调查者回答包，请按每包香烟20支计算；如果是抽烟丝自制的香烟，按照烟袋锅或者支计算；如果是雪茄，则每半支计算为一支）

I9.8.3 您现在是否已经戒烟了？（即停止吸烟半年及以上）

1□. 是，已戒烟 _________年 2□. 否

**I9.9**和您一起生活或者工作的人中有人吸烟吗？

1□. 是 2□. 否（跳至I9.10）

I9.9.1 您暴露被动吸烟（接触吸烟者呼出的烟雾超过15分钟/天）的频率是

1□. 几乎每天 2□. 平均≧3天/周 3□.平均1-3天/周 4□.平均<1天/周 5□.无

**I9.10**您是否有饮酒历史（每周至少1次）

1□. 是 □ 2□. 否（跳至I9.16）

**I9.10.1** 您饮酒 年

**I9.10.2**您饮酒频率是

1□. 每天或几乎每天 2□.3-4次/周 3□. 1-2次/周

**I9.10.3**您在大多数场合选择喝什么酒？(单选)

1□. 白酒 两/次

2□. 红酒 _____两/次

3□. 啤酒 _____瓶/次（250ml,及小瓶）

**I9.10.4**如已戒酒（即停止饮酒半年及以上），您戒酒 年

**I9.16** 您最近一个月进行有规律的锻炼吗？ 1□. 是 2□.否（跳至I9.23）

I9.16.1最常锻炼方式是

1□.走路 2□. 跑步 3□. 游泳 4□. 球类运动 5□. 气功 6□. 太极 7□.其他 _____

I9.16.2平均每周锻炼次数为_____次

I9.16.3平均每次锻炼的时间为_____分钟

**I9.23** 您过去两周内是否有病伤？ 1□.是 2□.否（跳至I9.24）

I9.23.1 您过去两周内是否因病伤就诊过？ 1□.是（跳至I9.23.3） 2□.否

I9.23.2 有伤病未就诊的原因？

1□.自感病轻 2□.经济困难 3□.就诊麻烦 4□.无时间 5□.交通不便 6□.无有效措施 7□.其它原因

I9.23.3过去两周内，因为该病伤第一次就诊是在下列哪类医疗机构？

1□.诊所/村卫生室 2□. 乡镇卫生院 3□. 社区卫生服务中心

4□. 县区属卫生机构 5□. 省辖市/地区/直辖市区属卫生机构

6□. 省/自治区/直辖市属及以上卫生机构 7□. 其它（请注明）____

I9.23.4 过去两周看病共花费了多少钱(包括已报销或能够报销的)？ 元

I9.23.5过去两周看病的花费中，自付多少钱？_______元

**I9.24** 2015年7月以来，您是否有医生诊断需要住院？ 1□.是 2□.否（跳至I9.25）

I9.24.1 2015年7月以来您是否住过院？ 1□.是（跳至I9.24.3） 2□.否

I9.24.2 您最近一次需住院而未住院的原因？

1□.没必要 2□.无有效措施 3□.经济困难 4□.医院服务差

5□.无时间 6□.无床位 7□.其它

I9.24.3您最近一次是在下列哪类医疗机构住院的？

1□.诊所/村卫生室 2□. 乡镇卫生院 3□. 社区卫生服务中心

4□. 县区属卫生机构 5□. 省辖市/地区/直辖市区属卫生机构

6□. 省/自治区/直辖市属及以上卫生机构 7□. 其它（请注明）____

I9.24.4这次住院的原因：

1□.疾病（请注明疾病名称）_______ 2□.损伤/中毒(请注明)____ 3□.康复

4□.计划生育 5□.分娩 6□.其他(请注明)____

I9.24.5 2015年7月以来院共花费了多少钱(包括已报销或能够报销的)？ 元

I9.24.6 2015年7月以来的住院花费中，自付多少钱？_______元

【CAPI】

If work==2，则被访者没有工作过，跳至访问员自填部分

if I4a.3.0==1，则被访者只有一份工作，不需要回答上一份及第一份工作情况

if I4a.4.0==1，则被访者只有两份工作，不需要回答上一份工作情况

**I9.25** 职业性有害因素接触史

| 工作类型 | 接触何种职业性有害物质（见编码34） | 是否定期体检 | 防护措施（可多选） | 防护用具佩  戴时间/工作日 | 防护效果 |
| --- | --- | --- | --- | --- | --- |
| 目前或最近工作 |  | 1□.是 2□.否 | ①②③④⑤⑥⑦□ | h/d |  |
| 上一份工作 |  | 1□.是 2□.否 | ①②③④⑤⑥⑦□ | h/d |  |
| 第一份工作 |  | 1□.是 2□.否 | ①②③④⑤⑥⑦□ | h/d |  |

备注

职业性有害因素包括：

1 .粉尘；如煤尘、石墨尘、石棉尘、水泥尘、陶瓷尘、电焊烟尘、铸造粉尘等。

2 .放射性物质类（电离辐射）如X 线、放射性同位素、放射线矿物、中子发生器。

3.化学类有毒或有腐蚀性的金属、气体、液体**（包括农药、化肥等）**

4 . 物理类职业危害，如高温、高气压、低气压、局部振动、紫外线、噪声、激光、电磁

辐射。

5 . 生物类职业危害，如炭疽杆菌、森林脑炎、布氏杆菌。

6 . 其它职业危害______________

7 . 以上均没

防护措施：①无 ②口罩 ③防护面罩 ④防护眼镜 ⑤工作服 ⑥防护手套 ⑦其他________

防护效果：①有效地保护 ②较好 ③一般 ④无效果 ⑤不舒适

**I9.26** 您是否曾有过工伤？ 1□.是 2□.否（跳至I9.27）

I9.26.1 最重的一次工伤发生时间：_______年_______月

I9.26.2受伤部位（可多填）_______

I9.26.3受伤的严重程度：

1□.导致残疾 2□.住院10 天及以上，未残疾

3□.住院1-9 天，未残疾 4□.就诊或休息一天 5□.其他（请注明）___

I9.26.4 治疗、康复（含假肢等辅助器具）的直接费用共计______元

其中工伤保险支付______元

个人支付______元

用人单位支付______元

I9.26.5 因为治疗、康复产生的间接费用或损失（包括本人误工费用、交通费、护理费用等）共计________元

I9.26.6是否经工伤保险部门认定为工伤？ 1□.是 2□.否

I9.26.7 是否进行了劳动能力鉴定？ 1□.是 2□.否(跳至I9.27)

I9.26.8 劳动能力鉴定结果:劳动功能障碍伤残______级

I9.26.9生活自理障碍等级：

1□.生活完全不能自理 2□.生活大部分不能自理

3□.生活部分不能自理 4□.无生活自理障碍

**I9.27** 您觉得自己有因为职业导致的疾病吗？1□.有，是什么？___ 2□.没有（结束本部分）

I9.27.1 您是否曾向劳动部门规定的职业病鉴定机构申请过职业病鉴定？

1□.是 2□.否

I9.27.2 您是否自己找其他的机构/医院鉴定过？ 1□.是 2□.否

I9.27.3 是否最终鉴定为法定职业病？ 1□.是 2□.否（跳至I9.27.6）

I9.27.4 职业病名称（准确填写职业病诊断鉴定书中的疾病名称）____________（建议提供法定职业病的目录，供调查者参考）

I9.27.5 发病时间：（指症状开始出现的时间）______年______月

确诊时间：（指最终确诊的时间） ______年______月

I9.27.6 是否进行了劳动能力鉴定？ 1□.是 2□.否（跳至I9.27.10）

I9.27.7 劳动能力鉴定结果

I9.27.8劳动功能障碍伤残______级

I9.27.9生活自理障碍等级：1□.生活完全不能自理 2□.生活大部分不能自理

3□.生活部分不能自理 4□.无生活自理障碍

I9.27.10 治疗、康复（含假肢等辅助器具）的直接费用共计______元

其中工伤保险支付______元

个人支付______元

用人单位支付______元

I9.27.11因为治疗、康复产生的间接费用或损失（包括本人误工费用、交通费、护理费用等）共计________元

**访问员自填**

**1、**在正式访问的时候，这份问卷是：（单选）

1.访问员访填 2.部分通过第三者翻译由访问员访填 3.其他（请注明）________

**2、**在访问过程中，被访者有没有表示过拒绝受访的意思呢？（单选）

1.开始 2.中间 3.最后 4.从未

**3、**在访问过程中，被访者是否表示不耐烦呢？（单选）

1.一直 2.有时 3.偶尔 4.从未

**4、**在访问过程中，被访者对访问员的信任程度如何？（单选）

1.很低 2.低 3.高 4.很高

**5、**在访问过程中，被访者是否应付？（单选）

1.大多数时间 2.有些时候 3.不像是 4.完全没有

**6、**被访者合作程度如何？（单选）

1.很不合作 2.不合作 3.合作 4.很合作

**7、**这份问卷访问所得的可靠程度如何？（单选）

1.很不可靠 2.不可靠 3.可靠 4.很可靠

**8、**访问时所用的语言是：（单选）

1.普通话 2.当地方言 3.其他（请注明）_________________

**9、**被访者的普通话熟练程度怎样？

1.非常流利 2.流利，略带地方口音 3.不太流利

4.听得懂但不会讲 5.既听不懂又不会讲

**10．**访问时访问员是否单独作业？（单选）

1.是 2.否，同另一同性访问员 3.否，同另一异性访问员

4.否，督导陪访 5.否，其他情形（请注明）__________________

**11**．您觉得被访者的长相怎样？（长相越好，评分越高）（）

1……2……3……4……5……6……7……8……9……10

**12、**访问时，有其他人在场吗？（复选）

1.没其他人在场 2.配偶在场 3.子女在场

4.父母/公婆在场 5.其他人在场

13、当天的天气情况如何

1.晴天 2.多云 3.阴天

4.小雨 5.中雨 5.大雨
